# Toward food-grade production of the *Glutamicibacter halophytocola* diamine oxidase using *Komagataella phaffii*

**DOI:** 10.1186/s13568-025-01990-7

**Published:** 2025-12-04

**Authors:** Anna Bechtel, Lucas Kettner, Jan Hessenberger, Kenny Vlassakakis, Lutz Fischer

**Affiliations:** Department of Biotechnology and Enzyme Science, Institute of Food Science and Biotechnology, University of Hohenheim, Garbenstr. 25, 70599 Stuttgart, Germany

**Keywords:** Diamine oxidase, Histamine intolerance, *Komagataella phaffii*, Methanol-free, Antibiotic-resistance-free

## Abstract

The diamine oxidase from *Glutamicibacter halophytocola* (DAO-GH) was recombinantly produced in *K. phaffii* using the constitutive glyceraldehyde-3-phosphate dehydrogenase promoter for methanol-free production. Firstly, *K. phaffii* clones were generated for intracellular and secretory DAO-GH production that still possessed antibiotic resistance due to the cloning procedure. For intracellular production, a maximum intracellular DAO activity of 15,404 nkat/L_culture_ was achieved in fed-batch bioreactor cultivations, while for secretory production, the highest extracellular DAO activity of 6,078 nkat/L_culture_ was achieved using the αMF signal peptide without its EAEA sequence. The intracellularly produced DAO-GH was partially purified in several purification steps with a yield of 80%, a purification factor of about 10 and specific DAO activity of 16.7 nkat/mg_protein_. The secretory DAO-GH production resulted in a specific DAO activity of 15.4 nkat/mg_protein_ already in the cell-free culture supernatant at the end of cultivation without further purification steps. The food industry aims to avoid the use of antimicrobial resistance in enzyme production, therefore, a new cassette plasmid with self-excisable antibiotic resistance markers was constructed for secretory DAO-GH production. The antibiotic-resistance-free *K. phaffii* clone generated with this plasmid achieved a maximum extracellular DAO activity of 4,770 nkat/L_culture_ in a fed-batch bioreactor cultivation. The DAO-GH obtained in this cultivation was spray-dried, resulting in a storable powder with 23 nkat/g_powder_ DAO activity and a water activity value of 0.12. This study demonstrated the secretion of recombinant DAO in a microbial host such as *K. phaffii* for the first time and provides a strategy for generating antibiotic-resistance-free *K. phaffii* clones.

**Supplementary Information:**

The online version contains supplementary material available at 10.1186/s13568-025-01990-7.

## Introduction

Diamine oxidases (DAOs; EC 1.4.3.22) catalyze the oxidative deamination of biogenic amines, such as histamine, tyramine, cadaverine and putrescine (Kettner et al. 2022a). DAOs usually have a homodimeric structure and employ protein-derived 2,4,5-trihydroxyphenylalanine-quinone (topaquinone, TPQ) and metal ions like copper and calcium as cofactors (Mcgrath et al. 2009; Kettner et al. 2022a). In the human body, DAO is expressed in the intestine, among other places, where it catalyzes the oxidative deamination of histamine to imidazole-4-acetaldehyde, hydrogen peroxide and ammonia (Schwelberger and Bodner 1997; Schwelberger et al. 1998). Histamine is present especially in fermented foods, such as sausages, sauerkraut and cheese, and can, thus, be taken up through food (Silla Santos 1996; Jarisch 2013). Histamine is rapidly degraded by DAO in the small intestine in healthy individuals (Maintz and Novak 2007). However, individuals with so-called “histamine intolerance” are unable to degrade histamine sufficiently due to an impairment in the DAO activity available. This deficiency leads to a surplus of histamine, which can trigger allergy-like symptoms (Maintz and Novak 2007).

One idea to deal with “histamine intolerance” is the oral intake of exogenous DAO to support the endogenous DAO in the human’s small intestine (Komericki et al. 2011; Yacoub et al. 2018; Izquierdo-Casas et al. 2019; Schnedl et al. 2019). Regarding this approach, it is essential to administer a sufficient amount of DAO activity, also to compensate for the activity loss due to proteolytic digestion by pancreatic peptidases (Kettner et al. 2022a, b; Bechtel et al. 2025). This highlights the importance of ensuring that DAO can be biotechnologically produced in adequate quantities. Another idea to deal with this intolerance is the degradation of histamine directly within the foods containing histamine prior to consumption (Naila et al. 2015; Kettner et al. 2022a; Moniente et al. 2022; Hou et al. 2024). The DAO must retain sufficient enzymatic activity under the conditions of the respective food for that approach to be effective (Kettner et al. 2022a). The use of DAO in fermented foods would primarily require the activity to be present at a low pH value due to lactic acid produced during fermentation and at low temperatures (Kettner et al. 2022a).

One DAO which could be suitable for both approaches (histamine degradation in the intestine and directly in foods), is the DAO from the Gram-positive bacterium *Glutamicibacter halophytocola* (DAO-GH) (Kettner et al. 2025). In a previous study, DAO-GH was recombinantly produced in the methylotrophic yeast *Komagataella phaffii* with a high activity yield of about 75 µkat/L_culture_ (Kettner et al. 2025). The DAO-GH showed broad pH and temperature profiles compared to other DAOs (Kettner et al. 2022a, 2025). The DAO-GH still showed about 5 and 20% of its maximum activity at a pH of 5 and 5.5, respectively, which could make it suitable for the degradation of histamine in fermented foods (Kettner et al. 2022a, 2025). The DAO-GH showed maximum activity at a pH of about 7 and more than 90% of its maximum activity at 37 °C when histamine was used as a substrate. Thus, DAO-GH would also be suitable for oral intake to degrade histamine in the small intestine (Kettner et al. 2022a, 2025).

The DAO-GH was produced intracellularly in *K. phaffii* in the study of Kettner et al. (2025), using the methanol-inducible alcohol oxidase 1 promoter (P_AOX1_). Utilizing P_AOX1_, protein expression in *K. phaffii* is repressed when carbon sources such as glucose and glycerol are used and strongly induced when methanol is used as a carbon source (Tschopp et al. 1987; Inan and Meagher 2001). However, methanol is flammable and toxic, which can be a considerable safety risk especially in large-scale bioreactor cultivations (Zhang et al. 2009). The constitutive glyceraldehyde-3-phosphate dehydrogenase promoter (P_GAP_) is an alternative promoter for methanol-free protein production in *K. phaffii* (Waterham et al. 1997; Zhang et al. 2009; Vogl and Glieder 2013). The strength of P_GAP_ depends on the carbon source and has been described to be highest when glucose was used, followed by glycerol and methanol (Waterham et al. 1997). In addition to P_GAP_, other constitutive promoters like P_TEF1_, P_PGK1_, P_ENO1_ and P_TPI1_ can be used for methanol-free protein production. However, the strength of most of these promoters has been described to be lower than that of P_GAP_ (Vogl and Glieder 2013). Using P_GAP_, expression levels comparable to those of P_AOX1_ were achieved, although this was highly dependent on the recombinant protein produced (Vogl and Glieder 2013; Rinnofner et al. 2022).

An advantage of the expression host *K. phaffii* is that it is able to produce recombinant proteins not only intracellularly but also extracellularly by secretion. *K. phaffii* secretes only small amounts of endogenous proteins, therefore, the recombinant protein typically represents the majority of the total protein present in the culture supernatant (Lin-Cereghino et al. 2007). Consequently, secretion of a heterologous protein can serve as an initial purification step, which can reduce downstream-processing costs.

*K. phaffii* has been granted “qualified presumption of safety” status by the European Food Safety Authority, and several proteins produced in this yeast have been designated as “generally recognized as safe” by the United States Food and Drug Administration, making it suitable for food-grade enzyme production (Ciofalo et al. 2006; Spohner et al. 2015; EFSA BIOHAZ Panel 2018). An important aspect of such food enzymes is that their use should not lead to the spread of antimicrobial resistance (EFSA CEP Panel 2021). Regarding complete elimination of this risk, antibiotic-resistance-free *K. phaffii* strains could be used to produce the food enzyme.

The aim of this study was the methanol-free production of DAO-GH with an antibiotic-resistance-free *K. phaffii*. Firstly, both intracellular and secretory production of DAO-GH was investigated with P_GAP_ in *K. phaffii* clones still possessing antibiotic resistance. In addition, a new expression cassette plasmid with self-excisable antibiotic resistance markers was constructed using the Golden Gate cloning method (Engler et al. 2008) to generate antibiotic-resistance-free *K. phaffii* clones.

## Materials and methods

### Chemicals, enzymes and kits

Tryptone (enzymatic digest from casein) was purchased from Merck KGaA (Darmstadt, Germany) and yeast extract from Acros Organics (New Jersey, USA). NaCl, sorbitol, glucose, agar–agar and ammonium hydroxide were purchased from Carl Roth GmbH (Karlsruhe, Germany). Peptone (from casein, enzymatic digest) and bovine serum albumin (BSA; A8022) were purchased from Sigma-Aldrich. Kanamycin sulfate and chloramphenicol were bought from Serva electrophoresis GmbH (Heidelberg, Germany). Zeocin was purchased from InvivoGen (San Diego, USA). The MoClo *Pichia* toolkit (provided by Volker Sieber; Addgene kit #1000000108) and the MoClo Yeast toolkit (provided by John Dueber; Addgene kit #1000000061) were obtained from Addgene (Watertown, USA) (Lee et al. 2015; Obst et al. 2017). Primers for polymerase chain reactions (PCR) were ordered from Biomers (Ulm, Germany). The pPpKC1 plasmid (accession number: 6602) was obtained from the *Pichia* Pool (Graz University of Technology, Institute of Molecular Biotechnology, Graz, Austria). The Q5® High-Fidelity DNA Polymerase, Q5® Site-Directed Mutagenesis Kit and restriction enzymes were purchased from New England Biolabs GmbH (Frankfurt am Main, Germany). The TaKaRa Ex Taq® DNA polymerase was bought from Takara Bio Inc. (Kusatsu, Japan). The Gene Ruler 1 kb Plus DNA Ladder was purchased from Thermo Fisher Scientific (Waltham, Massachusetts, USA). The Precision Plus Protein™ unstained protein standard (10–250 kDa) was bought from Bio-Rad Laboratories GmbH (Feldkirchen, Germany).

### Wildtype strains and media

*Escherichia coli* XL-1-blue was used for the cloning and propagation of plasmids. The cultivation of *E. coli* XL-1-blue was done in lysogeny broth medium at 37 °C containing the appropriate antibiotic (40 µg/mL chloramphenicol or 50 µg/mL kanamycin).

*Komagataella phaffii* ATCC 76273 (CBS7435 or NRRL Y-11430) was purchased from the American Type Culture Collection (Manassas, Virginia, USA). Cultivation of *K. phaffii* strains was done at 30 °C in different media depending on the experiment. *K. phaffii* transformants were selected on YPDS (yeast extract, peptone, dextrose, sorbitol) agar plates (Bechtel et al. 2025) containing 200 µg/mL Zeocin. *K. phaffii* strains were cultivated in yeast extract peptone methanol YPM (yeast extract, peptone, methanol) medium, as described by Li et al. (2017) to excise antibiotic resistance markers. The cultivation of *K. phaffii* strains in deep-well plates and cultivation tubes was done in YPD (yeast extract, peptone, dextrose) medium (Bechtel et al. 2025). Precultures for the bioreactor cultivations of *K. phaffii* clones were done in buffered minimal dextrose medium (Bechtel et al. 2025) with potassium phosphate buffer at pH 5 for intracellular and pH 6 for secretory DAO-GH production. The cultivation of *K. phaffii* clones in bioreactors was done in basal salts minimal medium with an initial glucose concentration of 40 g/L (BSM_glucose_) containing PTM_1_ trace salts solution, as described by Bechtel et al. (2025). The BSM_glucose_ medium was adjusted to pH 5 for intracellular and pH 6 for secretory DAO-GH production using 25% ammonium hydroxide.

### Plasmid construction

Plasmids were constructed using the MoClo Yeast and MoClo *Pichia* toolkits, according to Lee et al. (2015) and Obst et al. (2017), respectively. Three so-called cassette plasmids were constructed for DAO-GH production: a P_GAP_-DAO cassette plasmid (for intracellular DAO-GH production), a P_GAP_-αMF-DAO cassette plasmid (for secretory DAO-GH production) and a P_GAP_-αMF-DAO-SE cassette plasmid (for secretory DAO-GH production in antibiotic-resistance-free *K. phaffii* clone). The *dao-gh* gene used in this study was codon optimized for *K. phaffii* (Additional file Fig. S1) and synthesized by Invitrogen (Thermo Fisher Scientific, Waltham, MA, USA). Part plasmids used for cassette plasmid assemblies are listed in the Additional file 1 in Table S1 and cassette plasmids used in this study are shown in the Additional file 1 in Table S2. Part plasmids constructed in this study were generated by cloning the new sequence (e.g. FRT sequence) into the pYTK001 plasmid (entry vector), according to Lee et al. (2015). New sequences were amplified by PCR with Q5® High-Fidelity DNA Polymerase using the primers shown in the Additional file 1 in Table S3. The pPpKC1 plasmid (Ahmad et al. 2019) was used as template DNA for the amplification of FRT_1 and FRT_2 sequences and the Flippase (Flp) recombinase expression cassette. The 5’-type-specific overhang of the *dao-gh* gene in the pPTK-3-DAO-GH part plasmid (Kettner et al. 2025) was changed from TATG to TTCT by site-directed mutagenesis using the Q5® Site-Directed Mutagenesis Kit with the primers DAO-GH-fw and DAO-GH-rev (Additional file Table S3) to construct pPTK-3b-DAO-GH, which allowed the fusion to a signal peptide for secretory DAO-GH production. All of the part plasmids constructed were verified by sequencing.

The cassette plasmids P_GAP_-DAO and P_GAP_-αMF-DAO were constructed with the help of the previously constructed GFP-dropout cassette plasmid (Bechtel et al. 2024), according to Lee et al. (2015). The GFP-dropout-SE cassette plasmid with self-excisable antibiotic resistance markers (Additional file Table S2) was constructed to assemble the P_GAP_-αMF-DAO-SE cassette plasmid, according to Lee et al. (2015). The correct assembly of cassette plasmids was verified by restriction digestion and partial sequencing.

### Construction of recombinant *K. phaffii* clones

The P_GAP_-DAO and P_GAP_-αMF-DAO cassette plasmids as well as the P_GAP_-αMF-DAO-SE cassette plasmid were linearized using *Avr*II and transformed into the *K.* *phaffii* ATCC 76273 wildtype strain, as described by Bechtel et al. (2025). Transformants were selected on YPDS agar plates containing 200 µg/mL Zeocin.

The *K. phaffii* genomic DNA was isolated, as described by Bechtel et al. (2024), and integration of the cassette plasmids into the *K. phaffii* genome was verified by PCR using the TaKaRa Ex Taq® DNA polymerase, according to the manufacturer’s instructions. The primers P_GAP_-fw and tAOX1-rev (Additional file Table S3) were used to verify the genomic integration of the P_GAP_-DAO and P_GAP_-αMF-DAO cassette plasmids. The primers P_GAP_-fw and ZeoR-rev (Additional file Table S3) were used to verify the genomic integration of the P_GAP_-αMF-DAO-SE cassette plasmid. The PCR products were analyzed by agarose gel electrophoresis using the Gene Ruler 1 kb Plus DNA Ladder as a molecular size standard.

The antibiotic resistance markers (Zeocin and Kanamycin resistance markers) present on the integrated P_GAP_-αMF-DAO-SE cassette plasmid were then excised from the *K. phaffii* genome, as described by Li et al. (2017). Therefore, a sterile tube containing YPM medium was inoculated with a single *K. phaffii* colony and cultivated at 30 °C and 180 rpm. After 24 h, approximately 10 µL of the *K. phaffii* culture was streaked onto a YPD agar plate to obtain single colonies. The culture remaining in the tube was fed with 10 mL/L methanol and incubated for another 24 h at 30 °C and 180 rpm. After a total cultivation time of 48 h, the *K. phaffii* culture was streaked again onto a YPD agar plate, as described above, to obtain single colonies. After the incubation of the agar plates for about 48 h at 30 °C, single colonies were picked and transferred into 10 µL of sterile double distilled water (H_2_O_dd_). Afterwards, 5 µL of the latter was spotted on a YPD agar plate; the remaining 5 µL was spotted on a YPD agar plate containing 200 µg/mL Zeocin and incubated for about 24 h at 30 °C. Colonies which grew on YPD agar plates but not on those agar plates containing Zeocin indicated that the antibiotic resistance markers (ZeoR and KanR) were most likely excised from the *K. phaffii* genome. The genomic DNA was isolated and the excision of the markers was verified by PCR again using the primers P_GAP_-fw and ZeoR-rev (Additional file Table S3). No PCR product was expected if the markers had been excised.

### Screening of recombinant *K. phaffii* clones

Recombinant *K. phaffii* clones with integrated P_GAP_-DAO cassette plasmid were cultivated in YPD medium in deep well plates 96/2000 µL (Eppendorf AG, Hamburg, Germany), as described by Bechtel et al. (2025). The optical density (OD) reached at the end of the cultivation was measured at 595 nm (OD_595nm_) in a microtiter plate reader. Cells were disrupted in microtiter plates and investigated for their intracellular DAO activity (see below).

Recombinant *K. phaffii* clones with integrated P_GAP_-αMF-DAO cassette plasmid were cultivated in 1 mL with an initial OD_600nm_ of 1, and recombinant antibiotic-resistance-free P_GAP_-αMF-DAO *K. phaffii* clones were cultivated in 5 mL YPD medium in tubes with an initial OD_600nm_ of 0.1 at 30 °C and 180 rpm for 24 h. The OD reached at the end of the cultivation was measured at 600 nm (OD_600nm_) in cuvettes in a photometer. After cultivation, the samples were centrifuged (13,000 × g, 5 min, 4 °C) and the culture supernatants were analyzed for extracellular DAO activity (see below).

### Fed-batch bioreactor cultivations

Fed-batch bioreactor cultivations of recombinant *K. phaffii* clones were done in Multifors 2 bioreactors (1.4 L total vessel volume; Infors HT, Bottmingen, Switzerland), according to Bechtel et al. (2024). Briefly, the cultivation was done in BSM_glucose_ medium at pH 5 for intracellular and pH 6 for secretory DAO-GH production, with the latter pH chosen to ensure sufficient stability of DAO-GH, as its theoretical isoelectric point is 4.8 (https://web.expasy.org/compute_pi/). The initial fermentation volume in the bioreactor was 500 mL, which was inoculated with 10% (v/v) preculture. The culture was stirred at 1000 rpm, aerated with 1 – 2 vvm and supplemental oxygen was added as needed to maintain the pO_2_ above 20%. After the initially applied glucose was consumed an exponential glucose feed was started using a 400 g/L glucose solution containing 12 mL/L PTM_1_ trace salts solution. Feeding was started manually and then automatically controlled by the bioreactor software eve® (Infors HT, Bottmingen, Switzerland) with an exponential feeding profile. The exponential feed rate F(t) and initial feed rate F_0_ were calculated according to Looser et al. (2015). At the beginning of the fed-batch phase, the specific growth rate was set to 0.1 per hour. If no glucose accumulated during cultivation (verified by temporarily stopping feeding and monitoring the pO_2_ signal, as well as by glucose test strips), the feed rate was increased up to a specific growth rate of 0.15 per hour. During the cultivation, 5 mL samples were taken regularly and centrifuged (13,000 × g, 5 min, 4 °C). The culture supernatants were analyzed for extracellular DAO activity and cell pellets were washed with saline (0.9% (w/v) NaCl) and stored at − 20 °C until cell disruption to investigate the intracellular DAO activity. At the end of the cultivation, the *K. phaffii* culture was centrifuged at 8,000 × g at 4 °C for 15 min. The culture supernatant was analyzed directly for extracellular DAO activity and stored at 4 °C until concentration by cross-flow filtration and spray-drying.

### Disruption of *K. phaffii* cells

*K. phaffii* cell pellets were generally suspended in PIPES buffer (25 mM, pH 7.2) before the cells were disrupted using glass beads. After the cell disruption, samples were centrifuged and the cell-free extracts were investigated for intracellular DAO activity. Cell pellets obtained from deep well plate cultivations were disrupted in microtiter plates, as described by Bechtel et al. (2024). Cell pellets obtained during bioreactor cultivations were disrupted in Eppendorf tubes, according to Bechtel et al. (2024) with slight modifications. Therefore, 30% (w/v) cell suspensions were prepared and disruption was done using the TissueLyser II (Qiagen, Hilden, Germany) at a frequency of 30 Hz for 30 min. The supernatants obtained after centrifugation (13,000 × g, 5 min, 4 °C) were directly analyzed for DAO activity and protein content. An amount of 295 g K*. phaffii* bio wet mass was disrupted using the DYNO®-MILL KDL A (Willy A. Bachofen GmbH, Nidderau, Germany), according to Bechtel et al. (2024), for the purification of intracellularly produced DAO-GH. The supernatant obtained after centrifugation (10,000 × g, 45 min, 4 °C) was used for partial DAO-GH purification.

### Partial purification of intracellularly produced DAO-GH

The intracellularly produced DAO-GH was partially purified by precipitation of nucleic acids using polyethyleneimine (PEI), fractionated ammonium sulfate precipitation and hydrophobic interaction chromatography (HIC), according to Bechtel et al. (2025), with some modifications. All centrifugation steps were done at 10,000 rpm, 4 °C for 45 min. After the PEI precipitation of nucleic acids, an ammonium sulfate saturation of 25% was initially set in order to precipitate non-target proteins. Subsequently, the ammonium sulphate saturation was increased to 60% in order to precipitate the DAO-GH. The protein pellet obtained was dissolved in HIC binding buffer (25 mM sodium phosphate, pH 7, containing 1.3 M ammonium sulfate). Subsequently, DAO-GH (523 mL) was partially purified by HIC (column volume of 350 mL) using the Toyopearl Phenyl-650 M resin (Tosoh Bioscience, Tokyo, Japan).

### Concentration of *K. phaffii* culture supernatant using cross-flow filtration

The *K. phaffii* culture supernatant obtained after secretory DAO-GH production with the antibiotic-resistance-free clone was concentrated using a Vivaflow® 200 laboratory cross-flow cassette (Sartorius AG, Göttingen, Germany) equipped with a polyethersulfone membrane, which had a 10 kDa molecular weight cut-off. Before concentration, the membrane was covered with BSA by recirculating a 1% (w/v) BSA solution for about 15 min with a pump rate of 330 mL/min. The *K. phaffii* culture supernatant was concentrated about threefold using a pump rate of 330 mL/min, resulting in a flux of 13 mL/min. The concentrate was then desalted (fivefold) against sodium phosphate buffer (25 mM, pH 7) via diafiltration using the same Vivaflow® 200 cassette. Concentration and desalting were done at 4 °C.

### Spray-drying of DAO-GH

The concentrated and desalted *K. phaffii* culture supernatant obtained after secretory DAO-GH production with the antibiotic-resistance-free clone was spray-dried to obtain a storable DAO-GH preparation, according to Kettner et al. (2025). Briefly, a 20% (w/v) maltodextrin solution (in sodium phosphate buffer, 25 mM, pH 7) was mixed in a 1:1 ratio with the concentrated and desalted *K. phaffii* culture supernatant containing DAO-GH, resulting in a total volume of 350 mL. Spray-drying was done using the Büchi Mini Spray Dryer B-290 (BÜCHI Labortechnik AG, Flawil, Switzerland) with an inlet temperature of 170 °C and an outlet temperature of 90 °C (aspirator: 35 m^3^/h; pump rate: 2.6–2.9 mL/min; nozzle cap diameter: 1.5 mm). Some of the DAO-GH powder obtained was dissolved in potassium phosphate buffer (25 mM, pH 6.8) for the determination of residual DAO activity, which is described below. The water activity (aw) value was determined using the HygroPalm (Rotronic Messgeräte GmbH, Ettlingen, Germany).

### Protein analysis

The protein content of the enzyme samples was determined using BSA as a standard, according to Bradford (1976). Samples of the DAO-GH purification (intracellularly produced DAO-GH) and concentrated *K. phaffii* culture supernatant (secretory produced DAO-GH) were analyzed by sodium dodecyl sulfate (SDS)-polyacrylamide gel electrophoresis (PAGE) using 10% acrylamide-separating gels, according to Laemmli (1970). An amount of 5 µg protein was loaded onto each lane of the acrylamide gel. The Precision Plus Protein™ unstained protein standard (10–250 kDa) molecular weight marker was used as a reference. Protein bands were visualized by staining the gel using Coomassie Brilliant Blue R-250, according to Wang et al. (2024a).

### Determination of the DAO activity

The colorimetric DA-67 enzyme assay was used to determine the DAO activity, as described by Kettner et al. (2025). The enzymatic reaction was done with 1.35 mM histamine substrate solution in potassium phosphate buffer (25 mM, pH 6.8) at 37 °C. One katal (kat) of DAO activity was defined as the conversion of 1 mol substrate per second.

### Mass spectrometry analysis

Mass spectrometry analysis was done by the Mass Spectrometry Unit of the Core Facility Hohenheim at the University of Hohenheim (Stuttgart, Germany). The protein band analyzed was first excised from the SDS gel and proteins were in-gel digested, according to Shevchenko et al. (1996), with some modifications described by Senger et al. (2024). Furthermore, 1 ng/µL chymotrypsin (Promega, Madison, Wisconsin, USA) was used instead of trypsin in this study, and digestion was done at 25 °C overnight. A concentration of 50 mM NH_4_HCO_3_ was used in all steps in which the latter was employed.

Nano-LC–ESI–MS/MS experiments were done on an Ultimate 3000 RSLCnano system (Dionex, Thermo Fisher Scientific, Germany) coupled to an Orbitrap Exploris 480 mass spectrometer (Thermo Fisher Scientific, Germany) using a Nanospray Flex source (Thermo Fisher Scientific, Germany), as generally described by Senger et al. (2024). Chymotryptic peptides were injected directly into a precolumn (µ-precolumn C18 PepMap100, 300 µm, 100 Å, 5 µm × 5 mm, Thermo Fisher Scientific) and an analytical column (NanoEase M/Z HSS C18 T3, 1.8 µm 100 Å 75 µm × 250 mm column, Waters GmbH, Germany) maintained at a constant temperature of 35 °C. The gradient elution was carried out as described by Senger et al. (2024) starting in the first step with 2–55% solvent B within 30 min. The Orbitrap Exploris 480 and the Ultimate 3000 were operated under the control of XCalibur software (version 4.7.69.37) and Sii Xcalibur (version 1.8.0.530) (Thermo Fisher Scientific Inc., USA).

Mascot 2.6 (Matrix Science, UK) was used as a search engine for protein identification, as described by Senger et al. (2024). The spectra were searched against the DAO-GH protein sequence (including the signal peptide sequence) and the *Komagataella phaffii* protein database from Uniprot (https://www.uniprot.org/; March 2025). Mascot results were transferred to the Scaffold™ Software 4.10.0 (Proteome Software, USA) for validation.

### Statistical analysis

All experiments were done at least in duplicate and evaluated by determining the standard deviation with Excel (Microsoft, Redmond, USA). Data are presented as mean values with standard deviation.

## Results

### Construction and screening of recombinant *K. phaffii* clones for methanol-free DAO-GH production

**Fig. 1:**
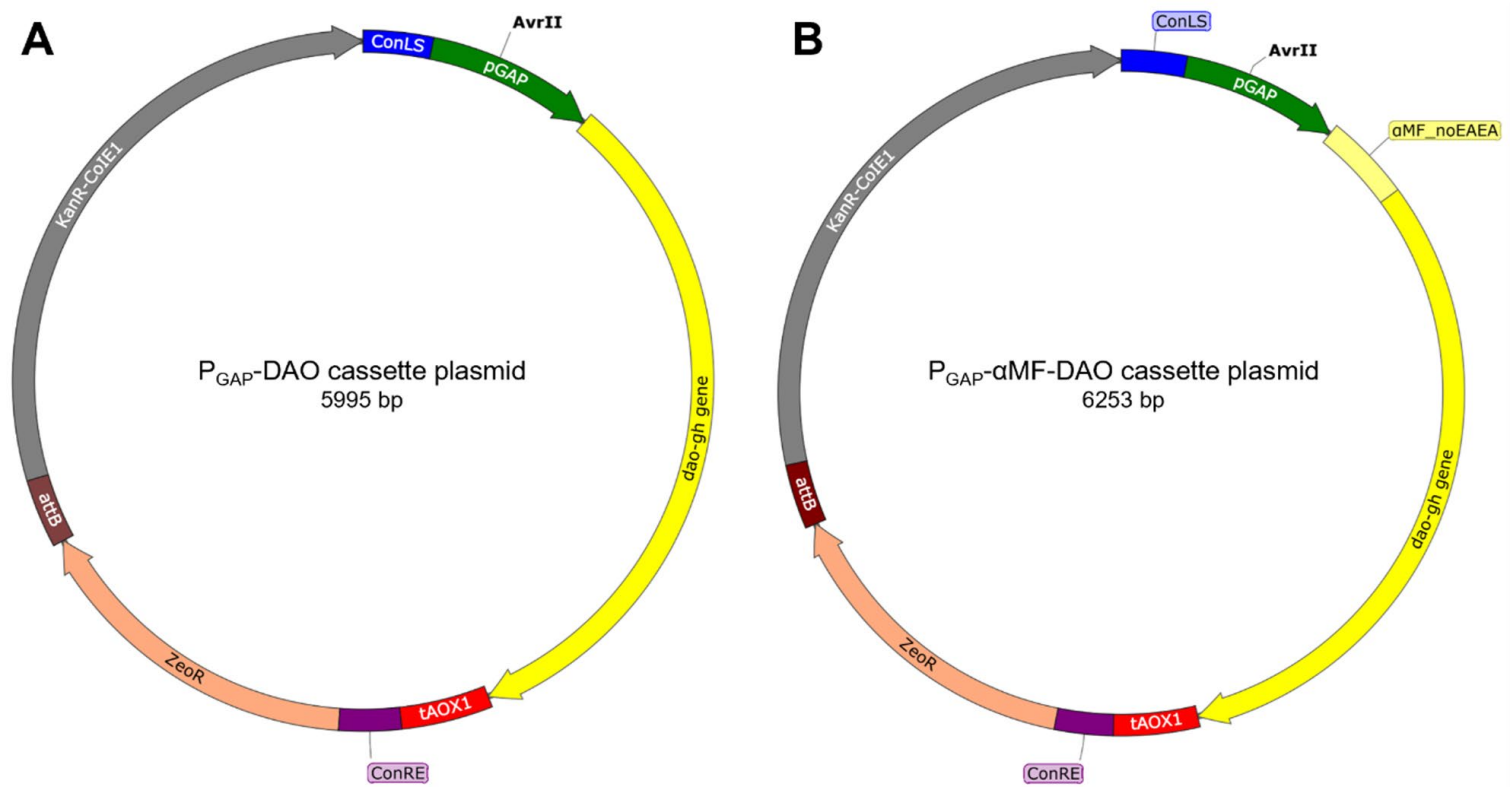
Cassette plasmids constructed for intracellular (**A**) and secretory (**B**) DAO-GH production with *K. phaffii*. pGAP = GAP promoter; αMF_noEAEA = αMF signal peptide without EAEA sequence; *dao-gh* gene = codon optimized *dao-gh* gene; tAOX1 = AOX1 terminator; ConLS and ConRE = assembly connectors; ZeoR = Zeocin resistance gene; *attB* = BxbI recognition site, KanR-CoIE1 = Kanamycin resistance gene and *E. coli* origin of replication

Cassette plasmids containing the P_GAP_ for methanol-free DAO-GH production were constructed for both intracellular and secretory DAO-GH production (Fig. 1), linearized within the promoter region and integrated into the genome of *K. phaffii* (Additional file Fig. S2). Regarding secretory production, the α-mating factor signal peptide without its native EAEA sequence (αMF_noEAEA) was used to direct DAO-GH into the secretory pathway.

Ten recombinant *K. phaffii* clones were generated for each construct and investigated for either intracellular or extracellular DAO activity. Clone 7 among the recombinant *K. phaffii* clones for intracellular DAO-GH production showed the highest intracellular DAO activity, with 151 ± 22 nkat/L_culture_/OD_595nm_ (Additional file Fig. S3). Clones 11 and 13 among the recombinant *K. phaffii* clones for secretory DAO-GH production showed the same extracellular DAO activity with 4.4 ± 0.1 nkat/L_culture_/OD_600nm_, which was the highest among the ten clones tested (Additional file Fig. S4). The activities of intracellularly and secretory producing DAO-GH clones cannot be compared at this level due to different screening conditions (e.g. cultivation time, working volume). Clone 7 was used for intracellular and clone 11 for secretory DAO-GH production in the subsequent bioreactor cultivations.

### Intracellular DAO-GH production with *K. phaffii*

**Fig. 2:**
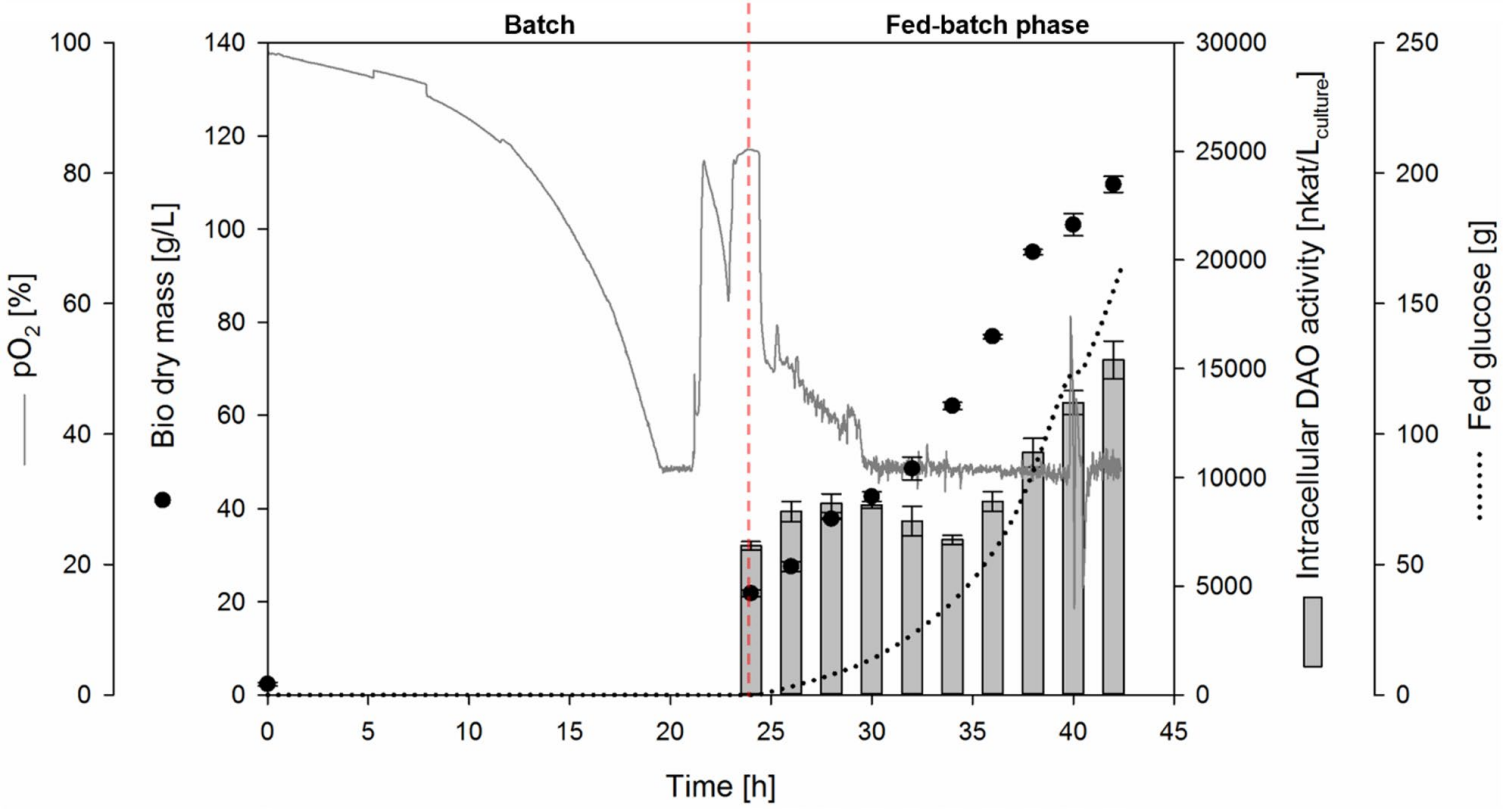
Fed-batch bioreactor cultivation of *K. phaffii* for intracellular DAO-GH production using P_GAP_. BSM_glucose_ medium, 0.5 L initial fermentation volume, 30 °C, pH 5. The pO_2_ signal is shown for one representative bioreactor from the biological duplicates. The pO_2_ profiles of both biological replicates are shown in Additional file Fig. S6

The carbon source glucose was completely consumed after approximately 21 h of bioreactor cultivation of *K. phaffii* with the integrated P_GAP_-DAO cassette plasmid (clone 7), as indicated by an increase in pO_2_ (Fig. 2). After approximately 24 h of cultivation, an exponential glucose feed was applied for intracellular DAO-GH production (Fig. 2). Since the glucose feed was started manually, there was a delay between the consumption of the carbon source and the start of the feed. The potential effect of this delayed feed start was not investigated in this study.

During fed-batch phase, a specific growth rate of 0.124 ± 0.002 per hour was achieved (Additional file Fig. S5).

The cultivation was stopped after 42 h because the maximum working volume of the bioreactor was reached. At this point, a maximum bio dry mass (BDM) of 110 ± 2 g/L and a maximum intracellular DAO activity of 15,404 ± 860 nkat/L_culture_ was achieved (Fig. 2), resulting in a specific cell activity of 141 ± 8 nkat per gram BDM. A specific productivity q_p_ of 15 ± 1 nkat/g_BDM_/h was achieved during the fed-batch phase.

After the bioreactor cultivation, 295 g K*. phaffii* bio wet mass (obtained from about 1.1 L cell culture) was disrupted, resulting in a total DAO activity of 16.9 ± 0.8 µkat (Table 1).

**Table 1:** Partial purification of DAO-GH after intracellular production in *K. phaffii*

	Volume [mL]	Protein [g]	EA [µkat]	Spec. EA [nkat/mg]	Yield [%]	Purification factor [−]
Crude	660	10.0 ± 0.1	16.9 ± 0.8	1.69 ± 0.08	100 ± 5	1.00 ± 0.05
PEI	600	7.2 ± 0.1	16.7 ± 0.4	2.32 ± 0.05	99 ± 2	1.37 ± 0.03
AS	523	4.5 ± 0.1	16.7 ± 0.4	3.72 ± 0.10	99 ± 3	2.20 ± 0.06
HIC	370	0.8 ± 0.04	13.4 ± 0.2	16.74 ± 0.26	79 ± 1	9.90 ± 0.15

Crude, Crude extract after cell disruption; PEI, Polyethyleneimine precipitation of nucleic acids; AS, Fractionated ammonium sulfate precipitation; HIC, Hydrophobic interaction chromatography; EA, DAO activity

DAO-GH was partially purified by the PEI precipitation of nucleic acids, fractionated ammonium sulfate precipitation and HIC (Additional file Fig. S7 chromatogram; Fig. S8 SDS-PAGE), resulting in a total activity of 13.4 ± 0.2 µkat with a specific activity of 16.74 ± 0.26 nkat/mg_protein_ (Table 1). Thereby, a yield of about 80% and a purification factor of about 10 was obtained.

### Secretory DAO-GH production with *K. phaffii*

**Fig. 3:**
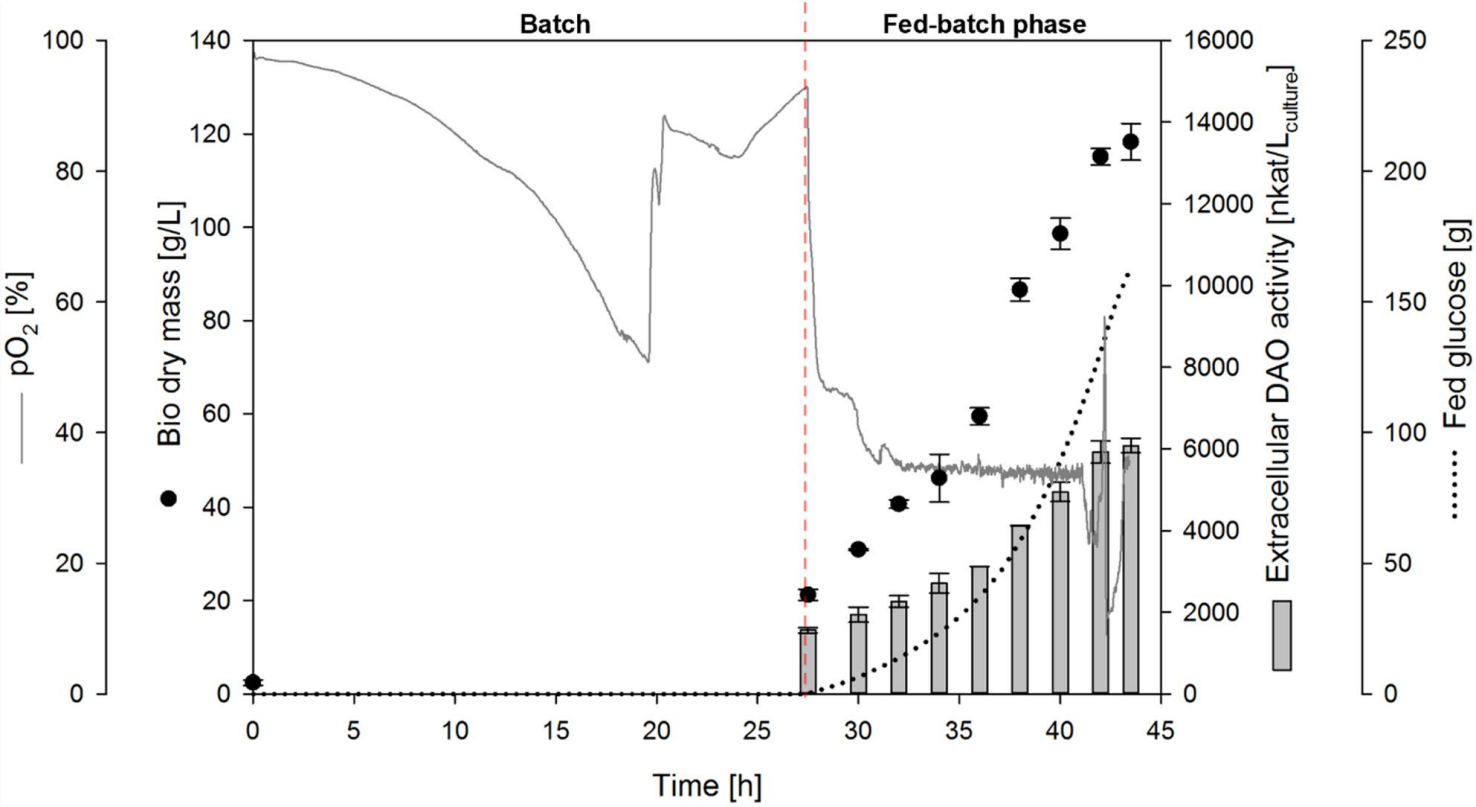
Fed-batch bioreactor cultivation of *K. phaffii* for secretory DAO-GH production using P_GAP_. BSM_glucose_ medium, 0.5 L initial fermentation volume, 30 °C, pH 6. The pO_2_ signal is shown for one representative bioreactor from the biological duplicates. The pO_2_ profiles of both biological replicates are shown in Additional file Fig. S10

When *K. phaffii* with the integrated P_GAP_-αMF-DAO cassette plasmid (clone 11) was cultivated for secretory DAO-GH production in the bioreactor, the carbon source glucose was completely consumed after approximately 20 h, as indicated by an increase in pO_2_ (Fig. 3). Due to manual feed initiation the exponential glucose feed was started after approximately 27 h of cultivation. During the exponential glucose feed (fed-batch phase), a specific growth rate of 0.150 ± 0.001 per hour was achieved (Additional file Fig. S9).

The highest BDM of 118 ± 4 g/L (corresponded to a bio wet mass of 454 ± 1 g/L) was reached at the end of the cultivation (43.5 h total cultivation time). At this point, the highest extracellular DAO activity was reached with 6,078 ± 179 nkat/L_culture_ (Fig. 3), resulting in a specific cell activity of 51.4 ± . 1.5 nkat/g_BDM_. A q_p_ of 7.4 ± 0.6 nkat/g_BDM_/h was achieved during the fed-batch phase. A specific DAO activity of 15.4 ± 0.1 nkat/mg_protein_ was obtained in the culture supernatant at the end of the cultivation, which was comparable to the specific DAO activity obtained after HIC purification of intracellularly produced DAO-GH (Table 1). An intracellular DAO activity of 2,840 ± 120 nkat/L_culture_ was determined at the end of the cultivation, resulting in a secretion efficiency of 68%.

### Construction of antibiotic-resistance-free *K. phaffii* clones for secretory DAO-GH production

**Fig. 4:**
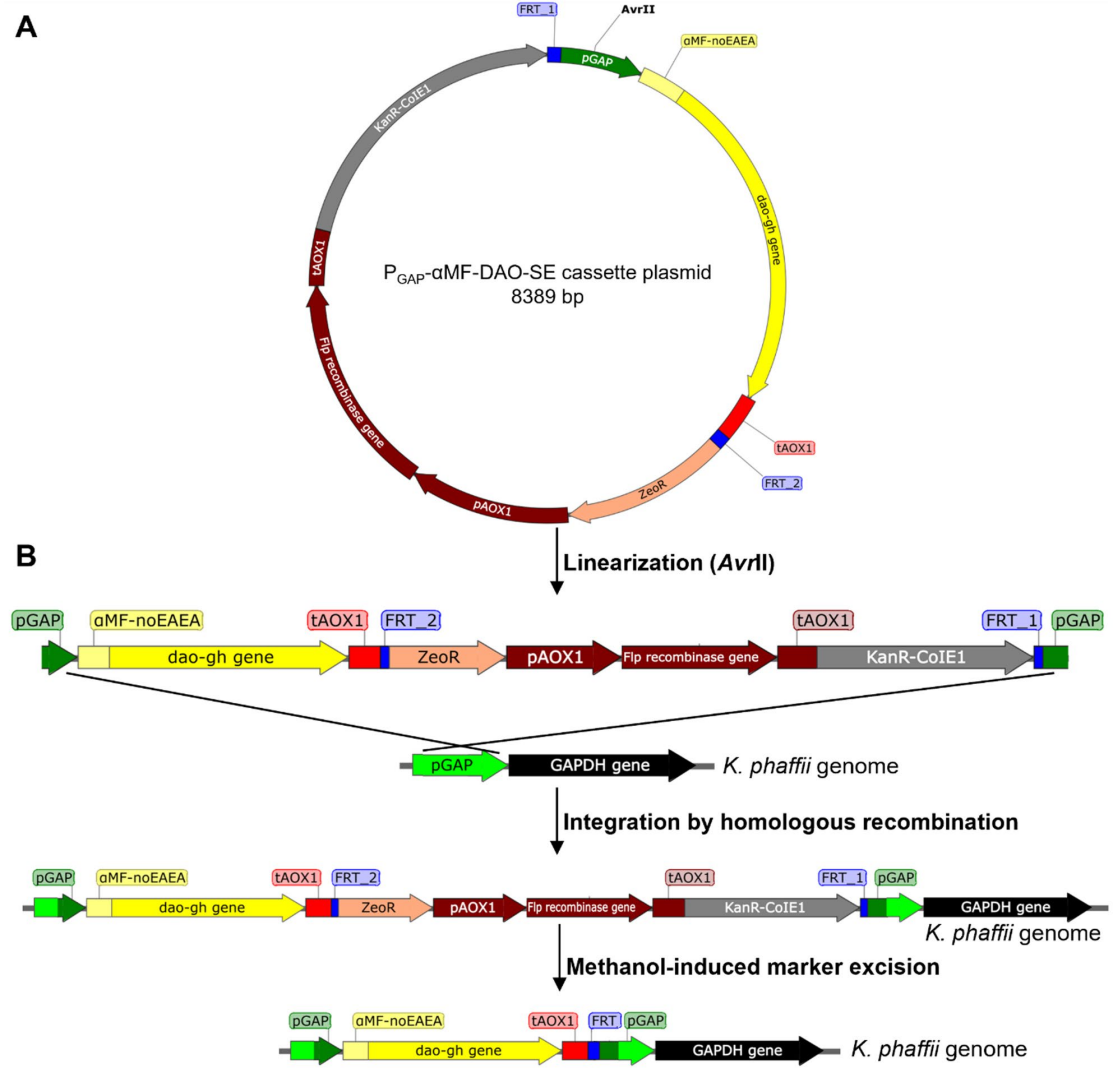
Construction of antibiotic-resistance-free *K. phaffii* clones for secretory DAO-GH production. Cassette plasmid with self-excisable antibiotic resistance markers (**A**). Linearization and integration of cassette plasmid into *K. phaffii* genome and methanol-induced expression of the Flp recombinase that catalyzes site-specific recombination between FRT sequences, leading to the excision of antibiotic resistance markers (**B**)

A new cassette plasmid with self-excisable antibiotic resistance markers was constructed for the generation of antibiotic-resistance-free DAO-GH secreting *K. phaffii* clones (Fig. 4A). The P_GAP_-αMF-DAO-SE cassette plasmid was linearized and integrated into the *K. phaffii* genome by homologous recombination (Fig. 4B; Additional file Fig. S11). The cassette plasmid contained a Zeocin resistance gene, therefore, *K. phaffii* transformants were first selected on agar containing Zeocin. Subsequently, the *K. phaffii* clones selected were cultivated in medium containing methanol to induce the P_AOX1_-driven expression of the Flp recombinase, an enzyme that catalyzes site-specific recombination between flippase recombination target (FRT) sequences. These FRT sequences were located upstream and downstream of the antibiotic resistance gene cassettes (ZeoR and KanR), enabling the excision of both the resistance gene cassettes and the Flp recombinase expression cassette from the *K. phaffii* genome through site-specific recombination (Fig. 4B).

Regarding the induction of Flp recombinase expression, the recombinant *K. phaffii* clones were cultured in medium containing methanol and spotted onto agar plates with and without Zeocin after 24 and 48 h to check for the excision of the antibiotic resistance markers (Additional file Fig. S12). In addition, marker excision was verified by PCR (Additional file Fig. S13). After the cultivation of recombinant *K. phaffii* clones in medium containing methanol for 24 h, the antibiotic resistance markers were excised in three out of ten clones (Additional file Figs. S12 and S13A). When the recombinant *K. phaffii* clones were cultured in medium containing methanol for 48 h, the antibiotic resistance markers were excised in all ten clones tested (Additional file Figs. S12 and S13B).

All antibiotic-resistance-free *K. phaffii* clones generated were checked for their extracellular DAO activity (Additional file Fig. S14). The highest extracellular DAO activity was obtained by clone 22 with 7.7 ± 1.0 nkat/L_culture_/OD_600nm_ (Additional file Fig. S14). Therefore, clone 22 was used for the subsequent bioreactor cultivation.

### Secretory DAO-GH production with antibiotic-resistance-free *K. phaffii*

**Fig. 5:**
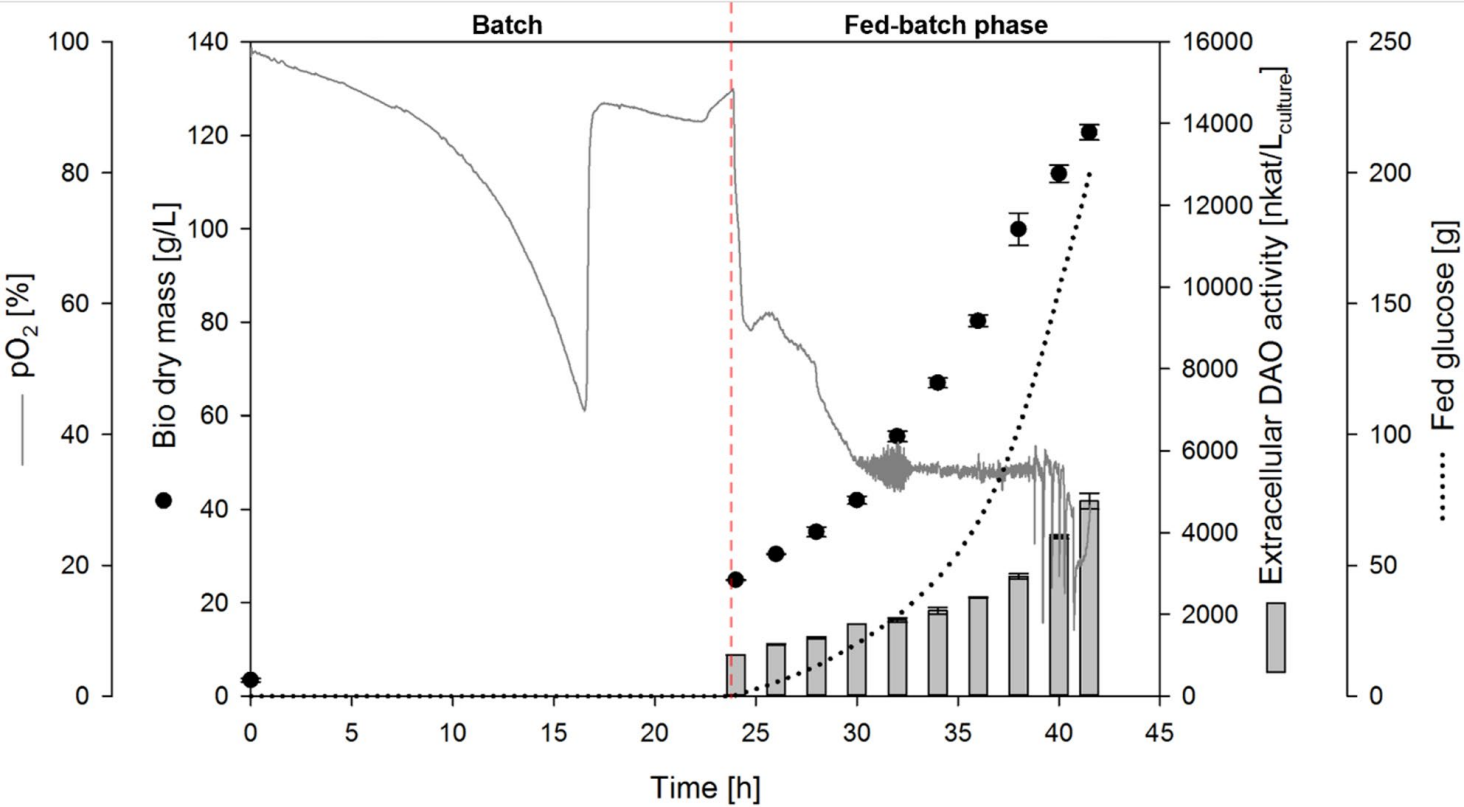
Fed-batch bioreactor cultivation of antibiotic-resistance-free *K. phaffii* for secretory DAO-GH production using P_GAP_. BSM_glucose_ medium, 0.5 L initial fermentation volume, 30 °C, pH 6. The pO_2_ signal is shown for one representative bioreactor from the biological duplicates. The pO_2_ profiles of both biological replicates are shown in Additional file Fig. S16

When the antibiotic-resistance-free *K. phaffii* clone (clone 22) was cultivated for secretory DAO-GH production, the carbon source glucose was completely consumed after approximately 17 h (Fig. 5). Due to manual feed initiation the exponential glucose feed was started after approximately 24 h of cultivation. During fed-batch phase, a specific growth rate of 0.1326 ± 0.0001 per hour was achieved (Additional file Fig. S15). Cultivation was stopped after about 42 h due to the volume limitation of the bioreactor vessel. The highest BDM was reached at the end of cultivation with 121 ± 2 g/L (corresponded to a bio wet mass of 446 ± 0.1 g/L) and the highest DAO activity with 4,770 ± 191 nkat/L_culture_ (Fig. 5), resulting in a specific cell activity of 40 ±  2 nkat/g_BDM_. A q_p_ of 5.2 ± 0.1 nkat/g_BDM_/h was achieved during the fed-batch phase. A specific DAO activity of 14.0 ± 0.6 nkat/mg_protein_ was obtained in the culture supernatant at the end of the cultivation.

**Fig. 6:**
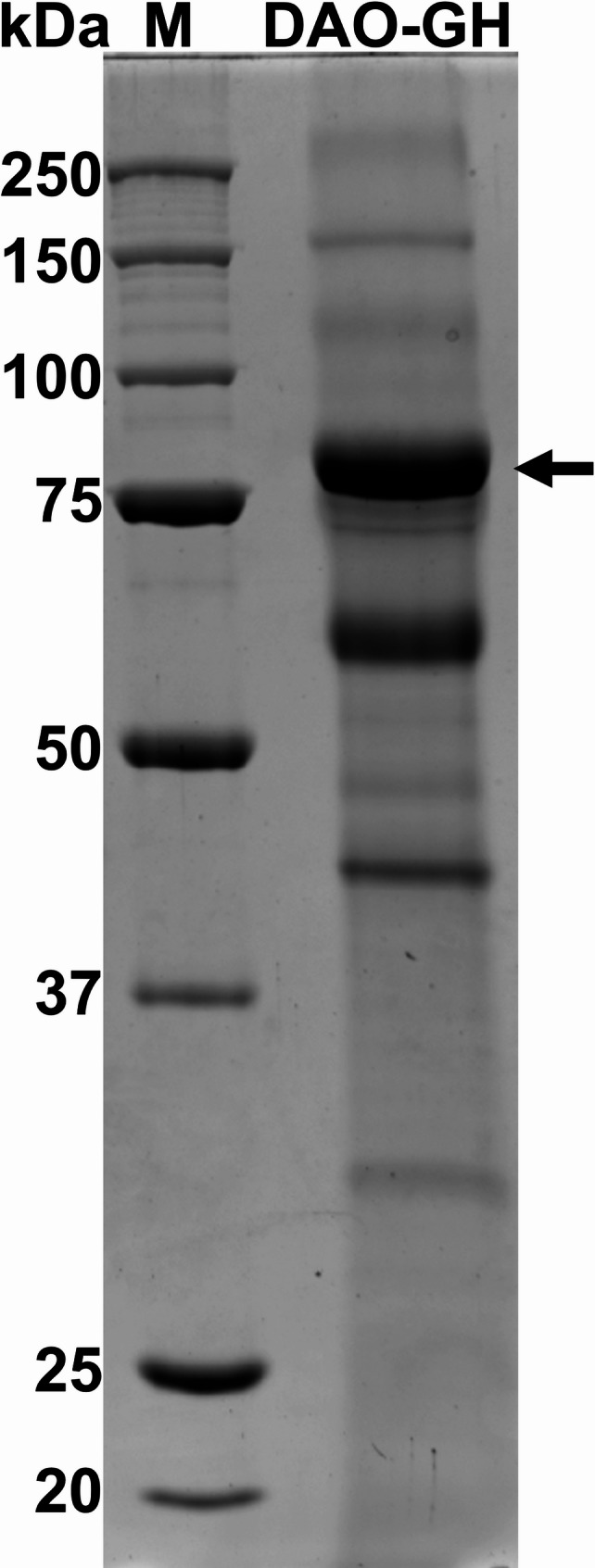
SDS-PAGE analysis of DAO-GH secreted by antibiotic-resistance-free *K. phaffii* clone. M = Precision Plus Protein™ unstained protein standard 10–250 kDa. The arrow indicates the protein band of the DAO-GH monomer (verified by mass spectrometry analysis)

From 840 mL of *K. phaffii* culture broth, approximately 500 mL cell-free culture supernatant were obtained by centrifugation, containing a total DAO activity of about 2,300 nkat. The culture supernatant was concentrated and desalted by cross-flow filtration (activity loss of about 30%), and then spray-dried to formulate a storable DAO-GH preparation. Before spray drying, the concentrated and desalted culture supernatant was analyzed by SDS-PAGE (Fig. 6). The analysis revealed a prominent band above 75 kDa, which was identified as DAO-GH by mass spectrometry (Additional file Fig. S17). In addition, mass spectrometry showed that the αMF_noEAEA signal peptide was cleaved from the DAO-GH, indicating that the DAO-GH was indeed secreted and not released by cell lysis. However, an intracellular DAO activity of 2,517 ± 88 nkat/L_culture_ was determined at the end of the cultivation, which corresponded to a secretion efficiency of 65% and showed that not all the DAO-GH could be secreted.

A total DAO activity of 1,581 nkat in a volume of 175 mL was spray-dried with maltodextrin as the carrier material, resulting in a yield of 40%. The specific DAO activity per gram powder was 23 ± 1 nkat/g_powder_, with an aw-value of 0.12.

## Discussion

The main focus of this study was to advance the food-grade DAO-GH production by investigating the methanol-free production of the enzyme in an antibiotic-resistance-free *K. phaffii*. In addition, the secretion of DAO-GH was investigated to facilitate downstream processing. The DAO-GH was discovered and recombinantly produced in *K. phaffii* in a previous study (Kettner et al. 2025). Thereby, DAO-GH was produced intracellularly by using the methanol-inducible P_AOX1_ for expression (Kettner et al. 2025). Since the intracellular production of DAO-GH has already been demonstrated in *K. phaffii*, methanol-free production was also initially investigated intracellularly in this study using the constitutive P_GAP_. Comparing intracellular DAO-GH production using P_AOX1_ (Kettner et al. 2025) and P_GAP_ (this study) as promoters, a 4.6-fold higher intracellular DAO activity of 70,185 nkat/L_culture_ was achieved when P_AOX1_ was used. However, the higher activity in the study by Kettner et al. (2025) was achieved after 90 h, whereas the activity in this study was achieved after 42 h of cultivation.

Comparing the partial purification of the intracellularly produced DAO-GH in this study with that of the study by Kettner et al. (2025), similar specific DAO activities with 16.7 nkat/mg_protein_ (this study) and 19.7 nkat/mg_protein_ (Kettner et al. 2025) were obtained. According to SDS-PAGE analysis, several proteins other than DAO-GH were still present in the purified samples from both studies. This partial purity should be sufficient for application in the food sector. Therefore, further optimization of the purification procedure was not the focus of this work.

The intracellular production of DAO-GH requires several downstream processing steps that increase production costs, therefore, the secretion of this enzyme was investigated using the production host *K. phaffii*. *K. phaffii* has been widely used in the past for the secretion of a broad range of recombinant enzymes (Nieto-Taype et al. 2020; Navone et al. 2021; Wang et al. 2023; Xue et al. 2024). However, the secretion of a recombinant DAO in a microbial host such as *K. phaffii* was demonstrated for the first time in this study.

The DAO-GH could be secreted in this study using the αMF signal peptide (derived from *Saccharomyces cerevisiae*) without its EAEA sequence. The αMF signal peptide and its derivatives are one of the most commonly used signal peptides in *K. phaffii* (Nieto-Taype et al. 2020; Navone et al. 2021; Zou et al. 2022). The cleavage of the EAEA sequence of the native αMF signal peptide by Ste13 peptidase during secretion can be inefficient (Julius et al. 1983; Brake et al. 1984; Ghosalkar et al. 2008), therefore, the αMF signal peptide without its EAEA sequence was used in this study and the correct cleavage was confirmed by mass spectrometry.

According to the EFSA regulations, the usage of food enzymes should not lead to the spread of antimicrobial resistance, which is associated with public health concerns (EFSA CEP Panel 2021). The industry’s desired possibility to eliminate the risk of antimicrobial resistance spread is to use antibiotic-resistance-free *K. phaffii* clones for the production of food enzymes. Therefore, an antibiotic-resistance-free *K. phaffii* clone was constructed for secretory DAO-GH production in this study. In addition, a second clone was generated for secretory DAO-GH production that still exhibited antibiotic resistance to use the same genetic elements as those for intracellular production, except for the signal peptide sequence required for secretory production. The extracellular DAO activity per liter culture as well as per gram BDM was about 1.3-fold higher for the clone holding the antibiotic resistance gene (6,078 nkat/L_culture_; 51 nkat/g_BDM_) than that of the antibiotic-resistance-free clone (4,770 nkat/L_culture_; 40 nkat/g_BDM_). The extracellular DAO activity per milligram protein was about 1.1-fold higher for the clone holding the antibiotic resistance gene (15.4 nkat/g_protein_) than that of the antibiotic-resistance-free clone (14.0 nkat/g_protein_). A reason for this difference in extracellular DAO activities between the antibiotic-resistant and antibiotic-resistance-free clones might be due to clonal variation (Aw et al. 2017). Furthermore, the clones did not grow in the bioreactor cultivations at the same specific growth rate, which could also have an influence on the product yield (Looser et al. 2015). The higher specific growth rate of the antibiotic-resistant clone may explain its approximately 1.4-fold higher specific productivity (7.4 nkat/g_BDM_/h) compared to the antibiotic-resistance-free clone (5.2 nkat/g_BDM_/h).

However, the extracellular DAO activities per liter culture were about 2.5 to threefold lower than the activity achieved by the intracellularly producing clone. Furthermore, up to 35% of the DAO activity in the DAO-GH secreting clones was still found inside the cells, indicating that there are still problems with the secretion of DAO-GH. The secretion of DAO-GH was investigated for the first time in this study without optimizing the recombinant clone and the production conditions. Future studies must, therefore, focus on improving the secretion of DAO-GH in *K. phaffii* to achieve the same or preferably higher product yields compared to intracellular production. There are numerous examples in the literature in which the secretion of recombinant proteins was improved in *K. phaffii* (Juturu and Wu 2018; Yu et al. 2020; Wang et al. 2023; Xue et al. 2024; Zhou et al. 2024). However, the optimal conditions must be found individually for each protein.

Antibiotic-resistance-free recombinant *K. phaffii* clones were generated in this study using a cassette plasmid with self-excisable antibiotic resistance markers. The cassette plasmid carrying the DAO-GH expression cassette and self-excisable antibiotic resistance markers was constructed using the MoClo Yeast and *Pichia* toolkits from Lee et al. (2015) and Obst et al. (2017), respectively. The Flp recombinase expression cassette and the corresponding FRT recognition sequences, which were necessary for excision of the antibiotic resistance markers, were integrated into the toolkits as new parts in the form of so-called ‘part plasmids’ in this study. Accordingly, these new part plasmids can be used to construct any new cassette plasmids with self-excisable antibiotic resistance markers with the MoClo Yeast and *Pichia* toolkits using the Golden Gate cloning method.

The strategy of first integrating plasmids into the *K. phaffii* genome using an antibiotic resistance marker for efficient selection and subsequently removing the marker from the genome was already applied in other studies (Li et al. 2017; Ahmad et al. 2019; Wang et al. 2024b). Ahmad et al. (2019) constructed plasmids with self-excisable markers for targeted gene knockouts in *K. phaffii*, utilizing the Flp recombinase with its corresponding recognition sequences for marker excision, which was also used in this study. After incubating *K. phaffii* clones in medium containing methanol for 24 and 48 h, the marker was excised in 50 and ≥ 95% of the clones, respectively (Ahmad et al. 2019). By comparison, the marker in this study was excised in 30 and 100% of the clones after 24 and 48 h incubation in medium containing methanol, respectively.

When the marker is excised by the Flp recombinase, an FRT sequence remains in the *K. phaffii* genome. If another cassette plasmid with a self-excisable marker is integrated into the same locus in the *K. phaffii* genome and its marker is excised using the Flp recombinase, recombination may occur between the newly introduced FRT sequences and the former FRT site left in the genome. Consequently, multiple integrations of the expression cassette into the same locus in the *K. phaffii* genome would not be possible with the Flp/FRT system used here.

Alternatively, the Cre/*loxP* system could be used to enable multiple integrations of the expression cassette into the same *K. phaffii* genomic locus. The Cre recombinase can catalyze the recombination between two mutant *loxP* sites, *lox71* and *lox66*, leaving the new *lox72* site behind, which displays significantly decreased binding affinity for the Cre recombinase preventing further Cre/*loxP*-mediated recombination (Carter and Delneri 2010). This Cre/*loxP* system has already been used for the excision of antibiotic resistance markers in the genome of *K. phaffii* (Li et al. 2017; Wang et al. 2024b). However, the *cre* recombinase gene (GenBank accession number: X03453.1) contains a *Bsm*BI restriction site which must be removed through silent mutation to enable its use in Golden Gate cloning with the MoClo Yeast and *Pichia* toolkits.

Another approach for antibiotic-resistance-free *K. phaffii* strain construction is the use of auxotrophic markers (Cregg et al. 1985). In this approach, multicopy integration of the expression cassette can also be challenging, as one copy of the auxotrophic marker is usually sufficient to recover prototrophy. However, multicopy integration can still be achieved by using defective auxotrophic markers (Seresht et al. 2013; Betancur et al. 2017). Alternatively, antibiotic-resistance-free *K. phaffii* strains can be generated using the CRISPR/Cas9 system, which enables marker-free genomic integration of the expression cassette (Gao et al 2022; García-Calvo et al 2025). However, efficient targeted integration is challenging in *K. phaffii*, as Cas9-introduced double-strand breaks are predominantly repaired by the non-homologous-end-joining (NHEJ) pathway, resulting in insertions or deletions of a few nucleotides (Näätsaari et al. 2012). Deletion of the *ku70* gene, which encodes a protein that plays a key role in the NHEJ repair mechanism, can promote homologous recombination, thereby increasing the efficiency of targeted integration (Näätsaari et al. 2012). However, NHEJ-deficient strains have disadvantages, such as reduced growth rates, which may limit their suitability for industrial applications (Carvalho et al. 2010; Näätsaari et al. 2012; Weninger et al. 2016).

After secretory production of the DAO-GH with the antibiotic-resistance-free *K. phaffii* clone, the DAO-GH was spray-dried to obtain a storable DAO-GH preparation. In the study by Kettner et al. (2025), the DAO-GH was spray-dried for the first time and the residual DAO activity of the DAO-GH powder was still at 93% after 12 weeks of storage at 20 °C. Compared to this study, the DAO activity per gram powder was 6.7-fold higher in the study by Kettner et al. (2025). However, a higher yield of about 80% was achieved after spray drying and the aw value of the DAO-GH powder was 0.23 (Kettner et al. 2025), whereas a yield of 40% and an aw value of 0.12 were achieved in this study. The spray-drying experiments in this study were done at laboratory scale and were primarily aimed at demonstrating the proof of principle. Optimization of formulation and drying parameters could be addressed in future studies.

This study demonstrated the methanol-free and secretory production of the DAO-GH in *K. phaffii* for the first time. Additionally, new DNA sequences were integrated into the existing MoClo Yeast and *Pichia* toolkits, enabling the construction of new cassette plasmids with self-excisable antibiotic resistance markers for the generation of antibiotic-resistance-free *K. phaffii* clones. However, the results also highlight the need for further optimization to enhance the production of DAO-GH in *K. phaffii*. Future studies should focus, among other aspects, on optimizing the fed-batch cultivation procedure. Furthermore, genetic stability is crucial for the industrial application of the generated *K. phaffii* clones and should therefore be investigated in future studies.

## Supplementary Information

Below is the link to the electronic supplementary material.

Supplementary Material 1

## Data Availability

All data generated or analyzed during this study are included in this published article [and its supplementary information files].

## Abbreviations

αMF_noEAEA: α-Mating factor signal peptide without EAEA sequence
aw: Water activity
BDM: Bio dry mass
BSA: Bovine serum albumin
BSM_glucose_: Basal salts minimal medium with glucose as carbon source
DAO: Diamine oxidase
DAO-GH: Diamine oxidase from *Glutamicibacter halophytocola*
*E. coli*: *Escherichia coli*
Flp: Flippase
FRT: Flippase recombination target
HIC: Hydrophobic interaction chromatography
H_2_O_dd_: Double distilled water
kat: Katal
*K. phaffii*: *Komagataella phaffii*
NHEJ: Non-homologous-end-joining
OD_595nm_: Optical density at 595 nm
OD_600nm_: Optical density at 600 nm
P_AOX1_: Alcohol oxidase 1 promoter
PCR: Polymerase chain reaction
PEI: Polyethyleneimine
P_GAP_: Glyceraldehyde-3-phosphate dehydrogenase promoter
P_GAP_-DAO: Cassette plasmid for intracellular DAO-GH production with P_GAP_
P_GAP_-αMF-DAO: Cassette plasmid for secretory DAO-GH production with P_GAP_ and αMF_noEAEA signal peptide
P_GAP_-αMF-DAO-SE: Cassette plasmid for secretory DAO-GH production with P_GAP_ and αMF_noEAEA signal peptide and self-excisable markers
q_p_: Specific productivity
SDS-PAGE: Sodium dodecyl sulfate–polyacrylamide gel electrophoresis
YPD: Yeast extract, peptone, dextrose
YPDS: Yeast extract, peptone, dextrose, sorbitol
YPM: Yeast extract, peptone, methanol
