# Supplementary material for: Toward food-grade production of the Glutamicibacter halophytocola diamine oxidase using Komagataella phaffii
Source: AMB Express. 2025 Dec 4;15:177. doi: 10.1186/s13568-025-01990-7 (PMC12708439; doi:10.1186/s13568-025-01990-7)
Supplement: Supplementary file 1 — Supplementary Material 1 [file 13568_2025_1990_MOESM1_ESM.pdf]

Toward food-grade production of the

Glutamicibacter halophytocola* diamine oxidase using *Komagataella phaffii

Anna Bechtel[1], Lucas Kettner[1], Jan Hessenberger[1], Kenny Vlassakakis[1], Lutz Fischer[1]*

[1]University of Hohenheim, Institute of Food Science and Biotechnology, Department of Biotechnology and Enzyme Science, Garbenstr. 25, 70599 Stuttgart, Germany

*Corresponding author:

E-mail address: lutz.fischer@uni-hohenheim.de

Tel.: +49 711 459 22311

Additional file 1

ATGGAACACTTGCACCCAACTACTGCTTTGGAAACTGCTCACCCTTTGGAGCAGATTACCTCCGA
AGAGATCCTGAGAACCAGAAAGATTTTGGCTGACGCCGGTTTGGTTGAGCAGACTACTAGATTTG
CCTACTTGGGTTTGCTGGACCCACCAAAGGATTTGTTGTACGCTGATGCTGGTACTGAGATCCCA
AGAAAGATCAGAGTCATGCTGTACGACCCAACTATCCCAAGATCCTTGGACATTACCATCTGTTTG
GCTTCCGCTGAGATTGAGTCCCAGAGAGAAATTGAAGCTGCTACCGAAGGTCAGGTCCCAGTTTT
GTTGGAAGAGTTCGACACCGTGGAAGAGATCTTGGCTAACGACGAAGGTTGGATCAAGGCTTTG
GCTTCTAGAGGTTTGTCCACCTCTCAGGTTAGAGTTGCTCCATTGTCTGCTGGTGTTTTCGACTAC
GAAAACGAGGAAGGTAAGAGACTGTTGAGAGGTTTGGGTTTCGTTCAGAACTCTCCAGAAGATCA
CGCTTGGGCTCACCCAATTGATAGATTGGTTGCCTTCGTCGACTTGGAGAACAGATGTGTCGACA
GATTGATTGACGACGGTCCAGTTCCAGTTCCTGACATCAACGGTAACTACACTGACCCACAAGTT
CACGGTGAGTTGAGAGATGACTTGAAGGCTATCGAGATCACTCAACCAGACGGTGCTTCTTTCAC
TGTTGACGGTAACCACTTGTCTTGGCTTGGTTGGGATTTGAGAGTTGGTTTCGACTCCAGAGAAG
GTTTGGTCTTGCACCAGATTCACCACACTCAAGACGGTACTAGAAGGCCATTGATTCACAGAGCT
TCCATTTCCGAGATGGTCGTTCCATACGGTGATCCATCTCCATACAGATCCTGGCAGAACTACTT
CGACACTGGTGAGTACTTGGTTGGTAGAGATGCCAACTCCTTGAAGTTGGGTTGTGACTGTTTGG
GTGAGATCCACTACATGTCCCCAATGGTTGCTGATGACTTCGGTAACCCAAGAGTCATCGACAAC
GGTATCTGTATCCACGAAGAGGACGCTGGTATTGGTTGGAAGCACACTGATGAATGGGCTGGTT
CTAACGAGGTCAGAAGAAACAGAAGATTGGTCGTTTCCTTCTTCACCACCGTTGGTAACTACGAC
TACGGTTTCTACTGGTACTTGTACCTGGACGGAACTATCGAGTTCGAGGCTAAGGCTACTGGTAT
CGTTTTCACTGCTGCTTTGCCACACAAGGGTTACGAATACGCTTCTGAGATTGCTCCAGGTTTGG
CTGCTCCATTTCACCAACATTTGTTCGGTGCCAGACTGGACATGATGATTGATGGTCATGCTAAC
GCCGTTGACGAGTTGGAGGTTGTTAGATTGCCAAAGTCTGAGGGTAACCCACACGGTAACGCTTT
CACTCAGTCCAGATTGAGATTGGGTACTGAGCAGCAGGCTGTCAGAGATGCTAATGCTGCTGCT
GGTAGAGTTTGGCAGGTTTCTAACCCAGACTCTTTGAACCACGTTGGTGAGCCAGTTGGTTACAC
TCTGTACCCACAAAACAACCCTACTTTGGCTATGGCTGACGACTCTTCCATTGCTGCTAGAGCTG
CTTTCACTAGACACGATTTGTGGGTGACTAGATTCGCTGAGGGTGAGTTGTATGCTGCCGGTGAT
TTCGTTAACAGAAACCCAGGTGGTGCTGGTTTGCCAGCTTTTGTTGAAGCTGACAGAGACATTGA
CGGTCAGGACATCGTTTTGTGGCATTCCTTTGGTCTGACTCACTTCCCAAGACCTGAGGACTGGC
CAATTATGCCAGTTGACACTGTTGGTTTCACCTTGAAGCCACACGGTTTCTTCAACGAGAACCCAA
TGTTGAATATCCCAGCCTCCACTTCTTCCCACTGTTCTATGCAAGCTCCAGAAACTGAAGGTCACT
GTGGTGCTTAA

Figure S1: ***Dao-gh*** **gene sequence codon optimized for** ***K. phaffii*****.**

Table S1: Part plasmids used in this study.

Plasmid	Type	Description/Parts	Reference
pYTK001	-	Part plasmid entry vector	(Lee et al. 2015)
pYTK002	1	ConLS (assembly connector)	(Lee et al. 2015)
pPTK-1-FRT	1	FRT_1 sequence	This study
pYTK047	234r	GFP dropout	(Lee et al. 2015)
pPTK002	2	P_{GAP}	(Obst et al. 2017)
pPTK006	3a	αMF_noEAEA	(Obst et al. 2017)
pPTK-3-DAO-GH	3	Codon optimized *dao-gh* gene with overhangs for intracellular production	(Kettner et al. 2025)
pPTK-3b-DAO-GH	3b	Codon optimized *dao-gh* gene with overhangs for extracellular production	This study
pPTK019	4	tAOX1	(Obst et al. 2017)
pYTK072	5	ConRE (assembly connector)	(Lee et al. 2015)
pPTK-5-FRT	5	FRT_2 sequence	This study
pYTK080	6	ZeoR	(Lee et al. 2015)
pPTK020	7	*attB* (BxbI recognition site)	(Obst et al. 2017)
pPTK-7-flippase	7	Flp recombinase expression cassette	This study
pYTK084	8	KanR-ColE1	(Lee et al. 2015)

Table S2: Cassette plasmids used in this study. The GFP-dropout cassette plasmid was constructed by Bechtel et al. (2024); the other four plasmids were constructed in this study.

Plasmid	Consisting of parts from part plasmids	Used for
P_{GAP}-DAO	pYTK002, pPTK002, pPTK-3-DAO-GH, pPTK019, pYTK072, pYTK080, pPTK020, pYTK084	intracellular DAO-GH production
P_{GAP}-αMF-DAO	pYTK002, pPTK002, pPTK006, pPTK-3b-DAO-GH, pPTK019, pYTK072, pYTK080, pPTK020, pYTK084	extracellular DAO-GH production
P_{GAP}-αMF-DAO-SE	pPTK-1-FRT, pPTK002, pPTK006, pPTK-3b-DAO-GH, pPTK019, pPTK-5-FRT, pYTK080, pPTK-7-flippase, pYTK084	extracellular DAO-GH production in antibiotic-resistance-free clones
GFP-dropout	pYTK002, pYTK047, pYTK072, pYTK080, pPTK020, pYTK084	construction of cassette plasmids
GFP-dropout-SE	pPTK-1-FRT, pYTK047, pPTK-5-FRT, pYTK080, pPTK-7-flippase, pYTK084	construction of cassette plasmids with self-excisable markers

Table S3: Primers used in this study. Type-specific overhangs for cloning of part plasmids, according to Lee et al. (2015), are underlined.

Primer	Sequence [5' – 3']	Used for
FRT_1-fw	GCATCGTCTCATCGGTCTCACCCTGCCTTTTGCTCACATGTGAAG	cloning of pPTK-1-FRT
FRT_1-rev	ATGCCGTCTCAGGTCTCACGTTGAGTAAGTTGGGCCTGATC	cloning of pPTK-1-FRT
FRT_2-fw	GCATCGTCTCATCGGTCTCAGCTGCTAACTCGGCCACTAGG	cloning of pPTK-5-FRT
FRT_2-rev	ATGCCGTCTCAGGTCTCATGTACTTTGGATGTTAGATCTGAAG	cloning of pPTK-5-FRT
Flippase-fw	GCATCGTCTCATCGGTCTCAGAGTAGATCTAACATCCAAAGACG	cloning of pPTK-7-flippase
Flippase-rev	ATGCCGTCTCAGGTCTCATCGGAGCTCTCACTTAATCTTCTGTACTC	cloning of pPTK-7-flippase
DAO-GH-fw	GTCGGTCTCATTCTGAACACTTGCACC	cloning of pPTK-3b-DAO-GH
DAO-GH-rev	TACGGTTATCCACAGAATCAG	cloning of pPTK-3b-DAO-GH
P_{GAP}-fw	GTCCCTATTTCAATCAATTGAAC	verification of genomic integration
tAOX1-rev	GCAAATGGCATTCTGACATCC	verification of genomic integration
ZeoR-rev	GACGAGGCAAGCTAAACTG	verification of genomic integration

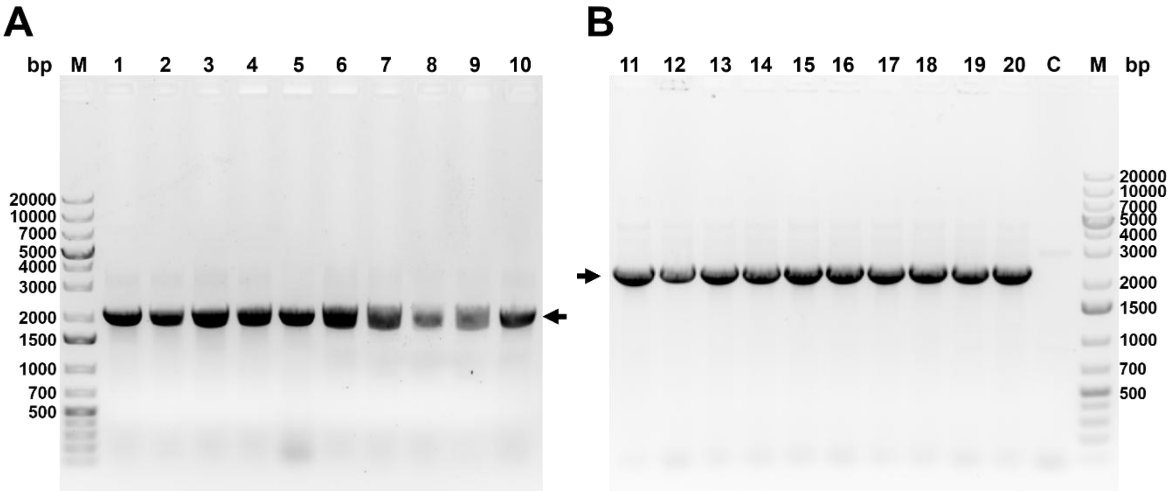

Figure S2: Verification of the integration of P$_{GAP}$-DAO (A) and P$_{GAP}$-*α*MF-DAO (B) cassette plasmids into the *K. phaffii* genome by PCR. 1 % (w/v) agarose gels. M = Gene Ruler 1 kb Plus DNA Ladder. C = control (*K. phaffii* ATCC 76273). 1–20 = recombinant *K. phaffii* clones. Arrows indicate the expected DNA bands for integration of the cassette plasmids into the *K. phaffii* genome.

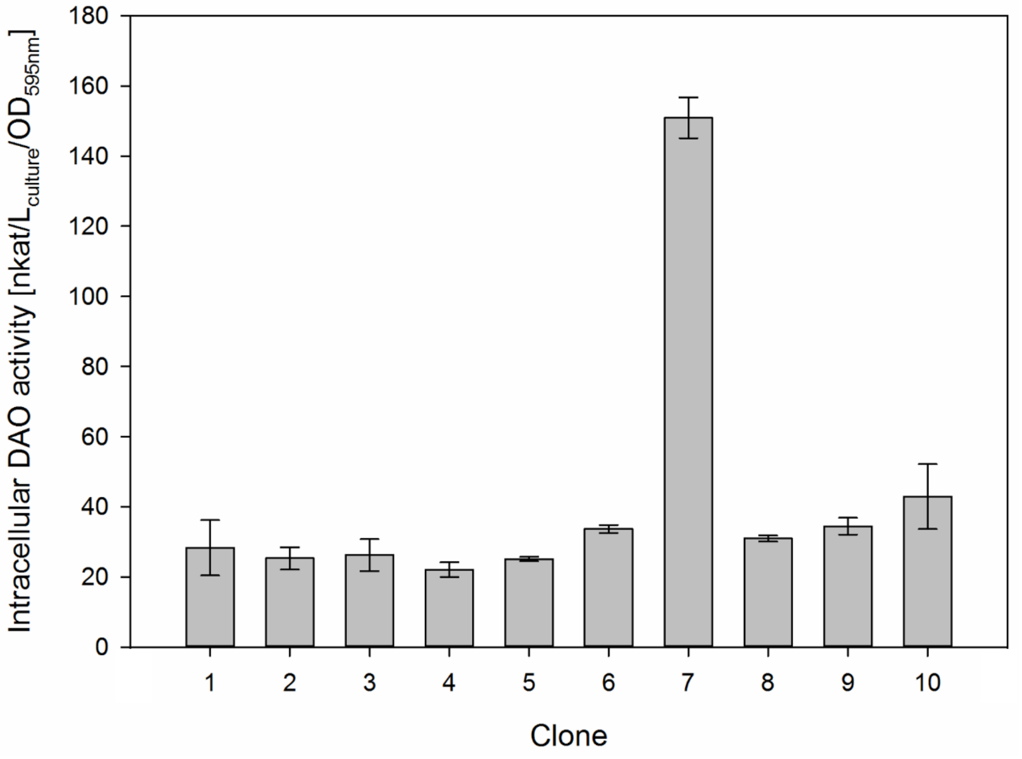

Figure S3: Investigation of the intracellular DAO activity of *K. phaffii* P$_{GAP}$-DAO clones. The cultivation was done in deep-well plates in a 500 µL working volume at 30 °C using YPD medium. The DAO activity was determined after 48 h of cultivation.

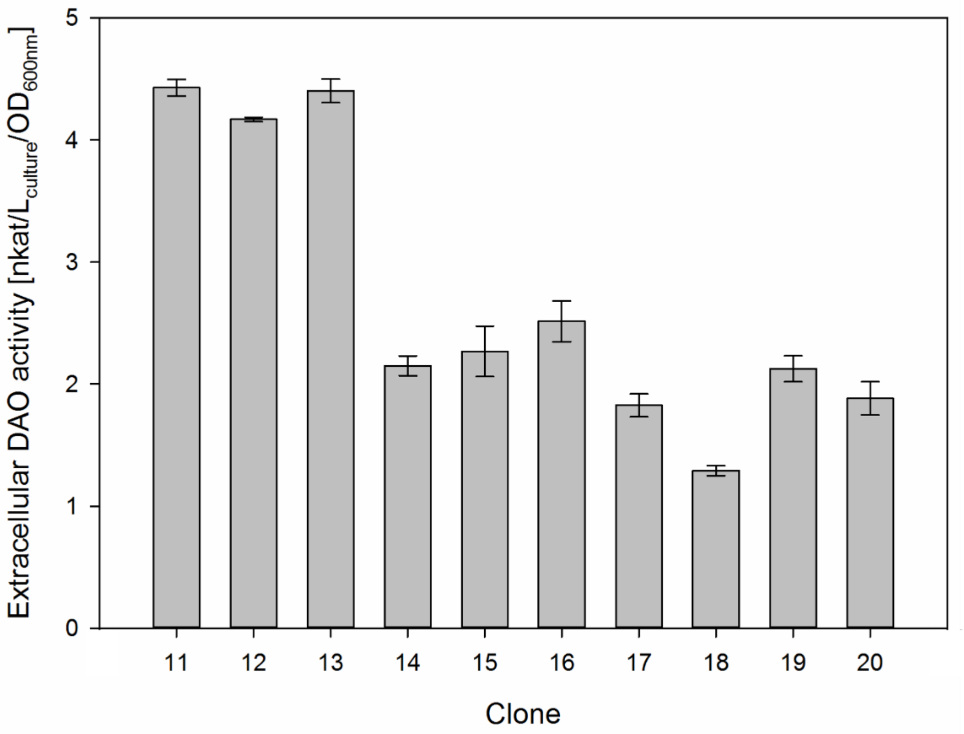

Figure S4: Investigation of the extracellular DAO activity of *K. phaffii* P$_{GAP}$-αMF-DAO clones. The cultivation was done in tubes in a 1 mL working volume at 30 °C using YPD medium. The DAO activity was determined after 24 h of cultivation.

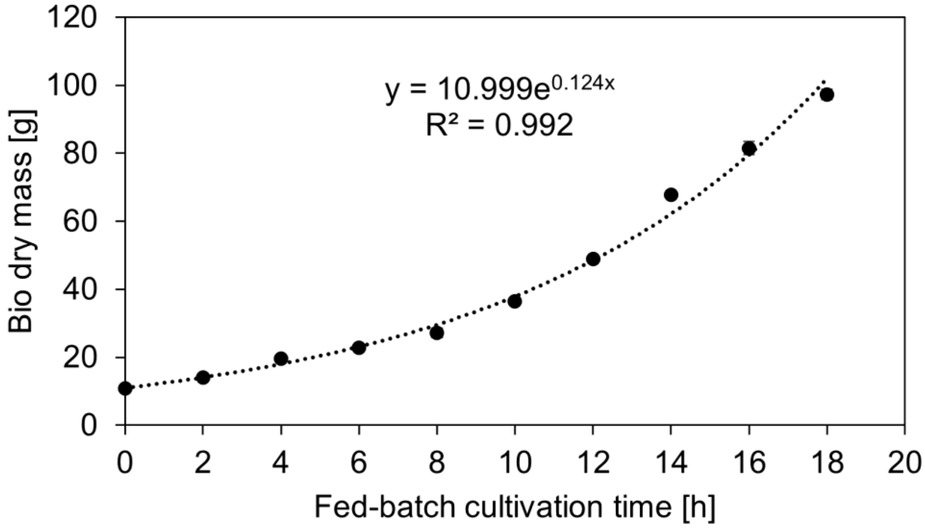

Figure S5: Total bio dry mass during the fed-batch bioreactor cultivation of *K. phaffii* P$_{GAP}$-DAO. BSM$_{glucose}$ medium, 0.5 L initial fermentation volume, 30 °C, pH 5. The specific growth rate was determined by fitting an exponential curve through experimental data.

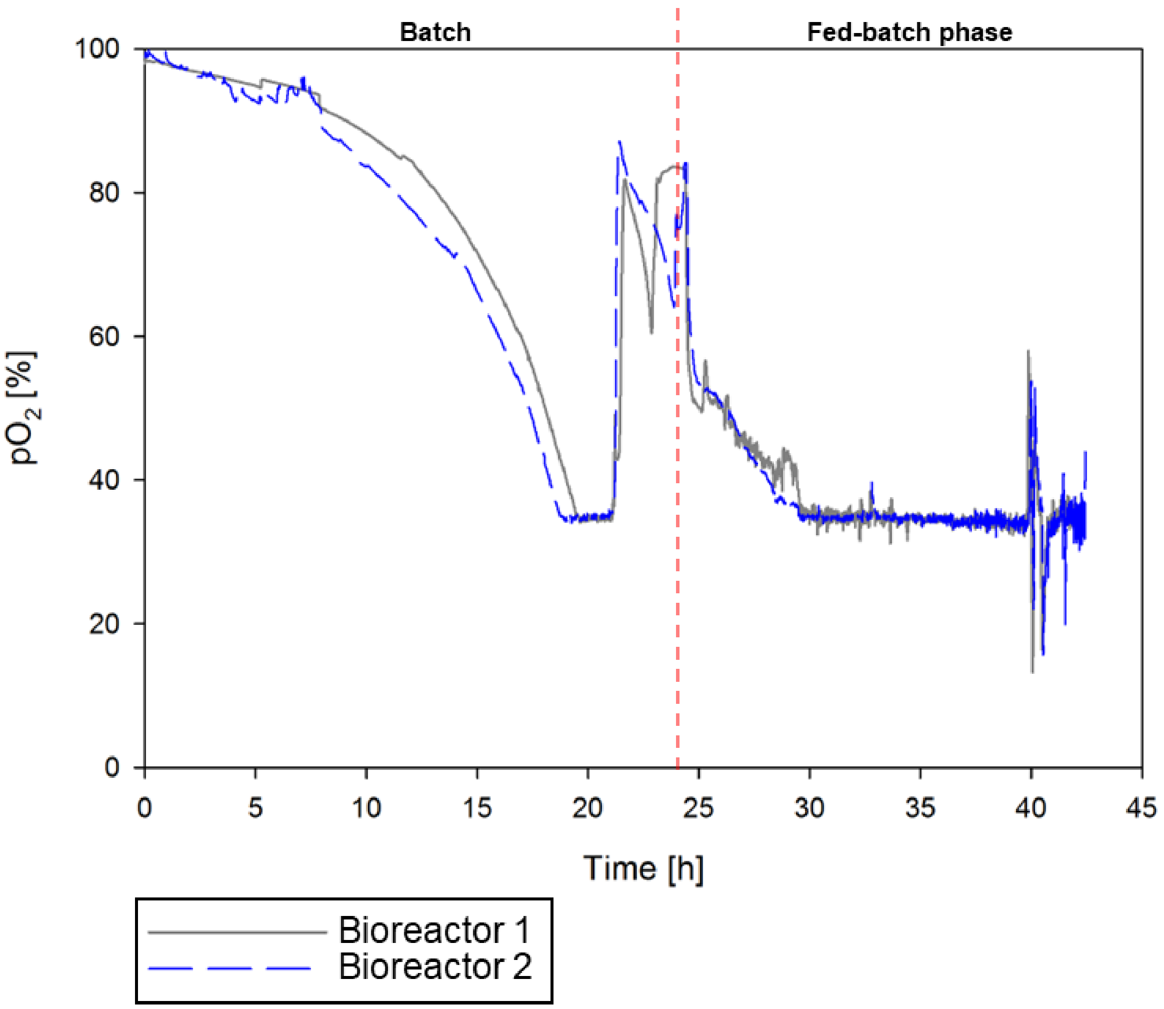

Figure S6: pO_2 profiles of biological duplicates during fed-batch bioreactor cultivations of *K. phaffii* for intracellular DAO-GH production. $BSM_{glucose}$ medium, 0.5 L initial fermentation volume, 30 °C, pH 5.

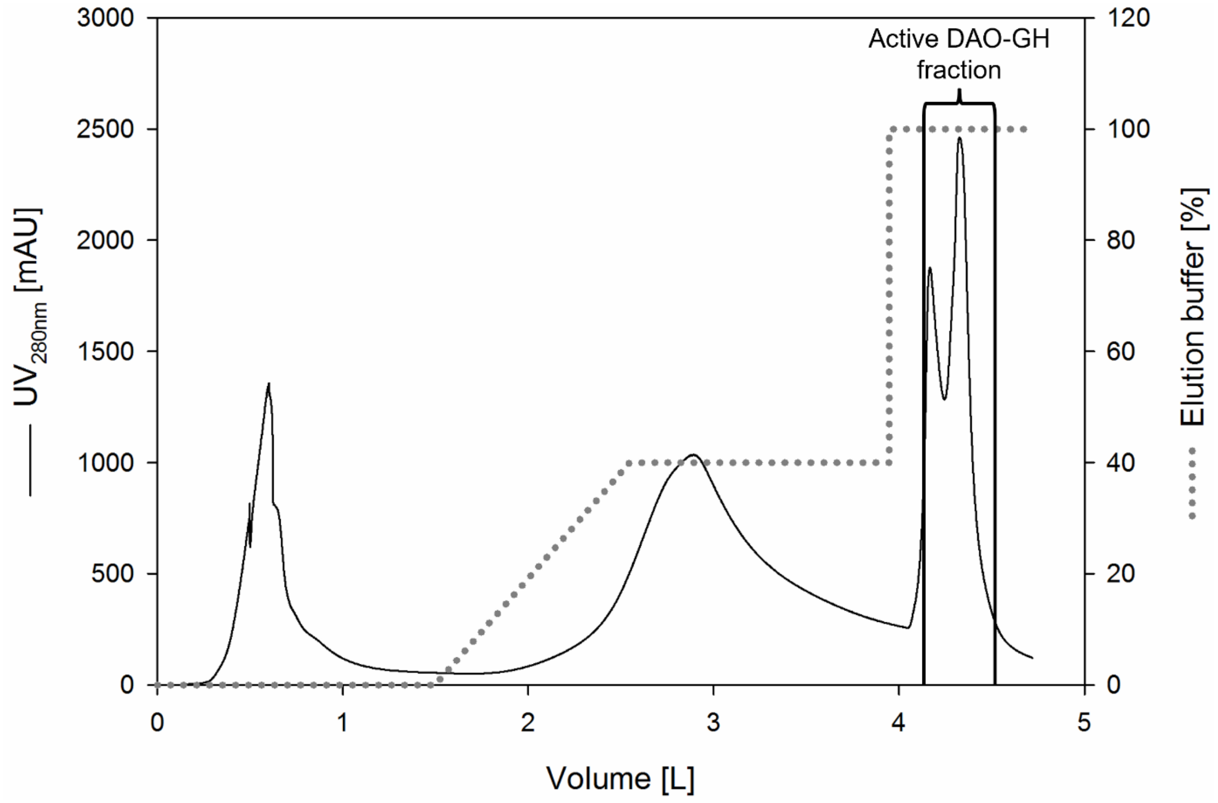

Figure S7: Chromatogram of the hydrophobic interaction chromatography of intracellularly produced DAO-GH. Column material: Toyopearl phenyl 650M (CV = 350 mL).

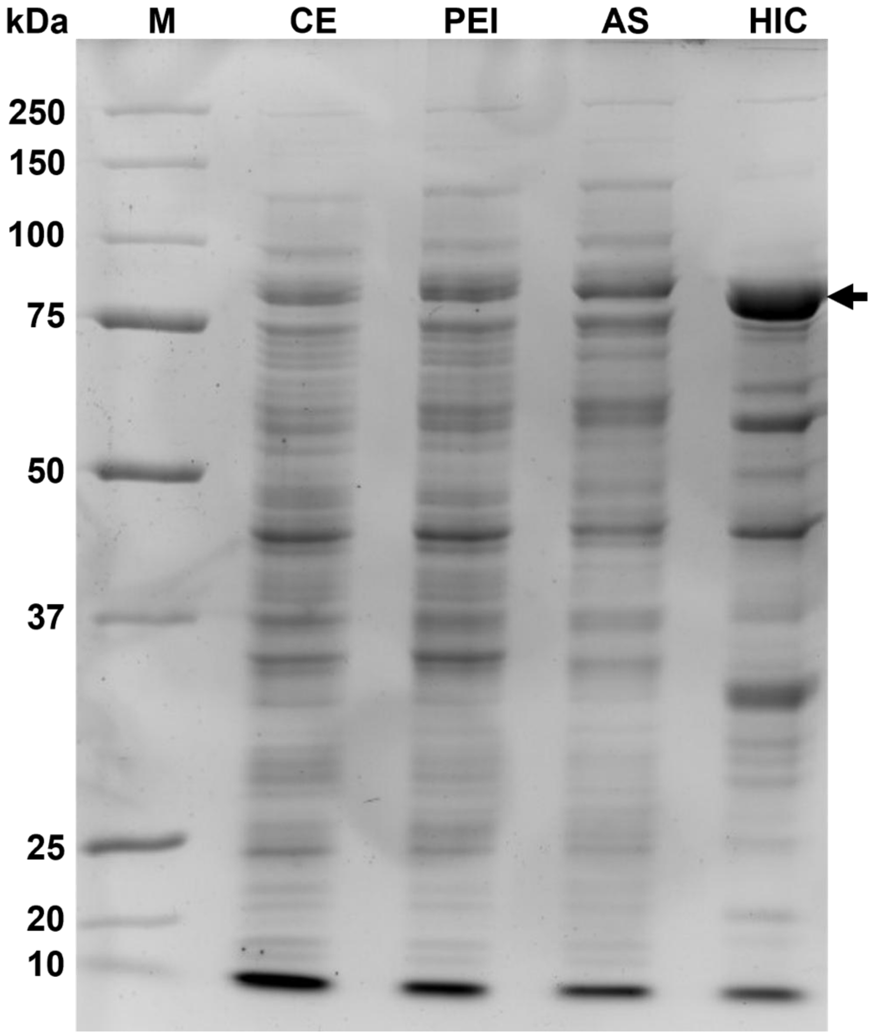

Figure S8: SDS-PAGE analysis of the DAO-GH purification. M = Precision Plus Protein™ unstained protein standard 10–250 kDa; CE = crude extract after cell disruption; PEI = polyethyleneimine precipitation of nucleic acids, AS = ammonium sulfate precipitation; HIC = hydrophobic interaction chromatography. The arrow indicates the expected band for DAO-GH.

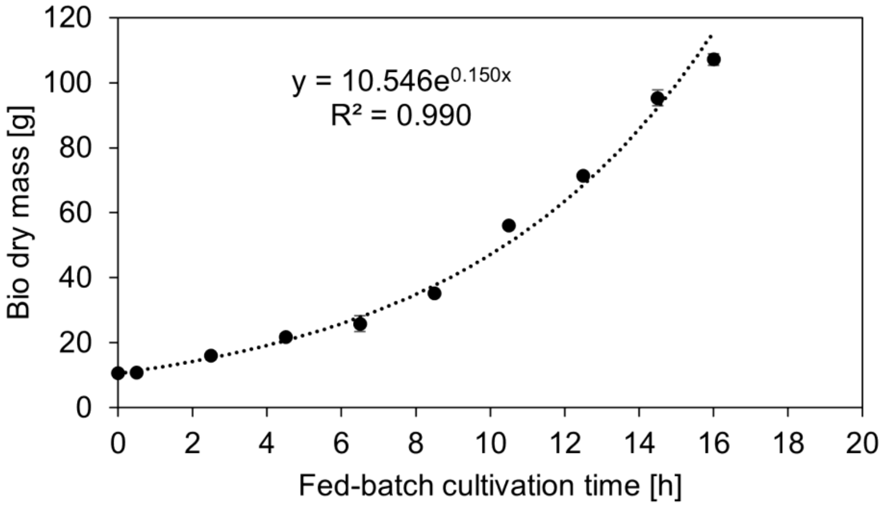

Figure S9: Total bio dry mass during the fed-batch bioreactor cultivation of *K. phaffii* P_{GAP}-αMF-DAO. $BSM_{glucose}$ medium, 0.5 L initial fermentation volume, 30 °C, pH 6. The specific growth rate was determined by fitting an exponential curve through experimental data.

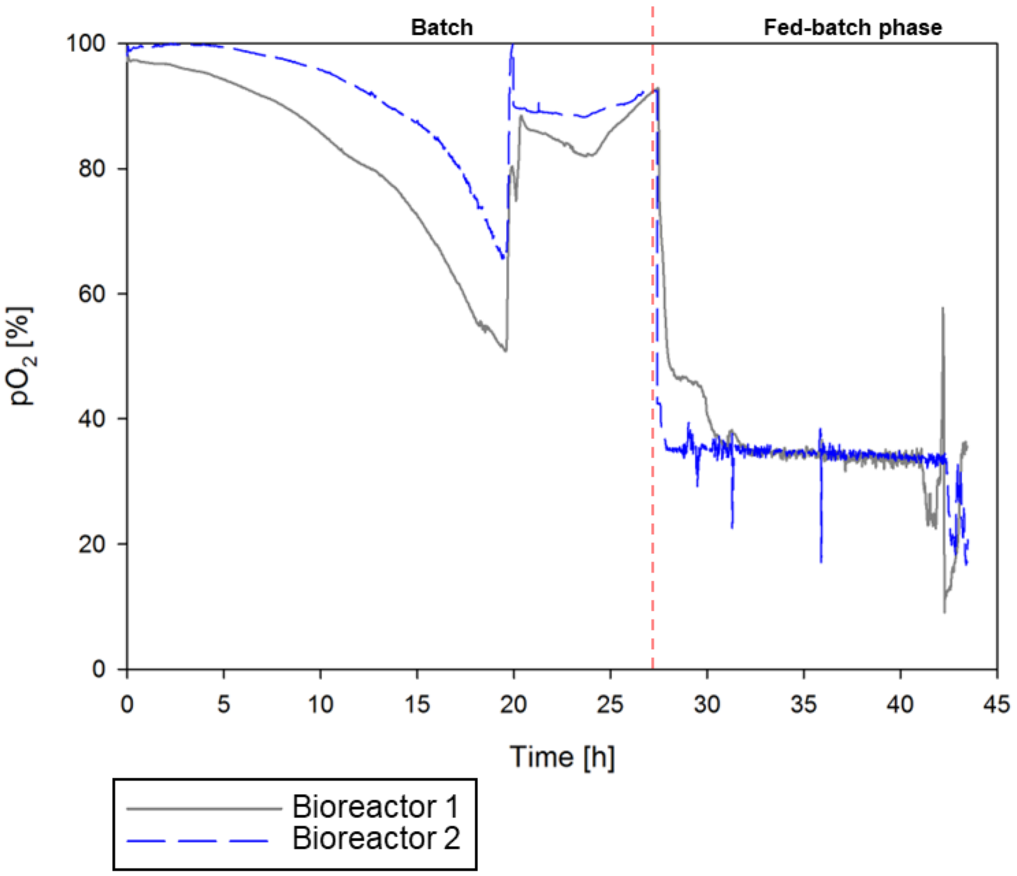

Figure S10: pO_2 profiles of biological duplicates during fed-batch bioreactor cultivations of *K. phaffii* for secretory DAO-GH production. $BSM_{glucose}$ medium, 0.5 L initial fermentation volume, 30 °C, pH 6.

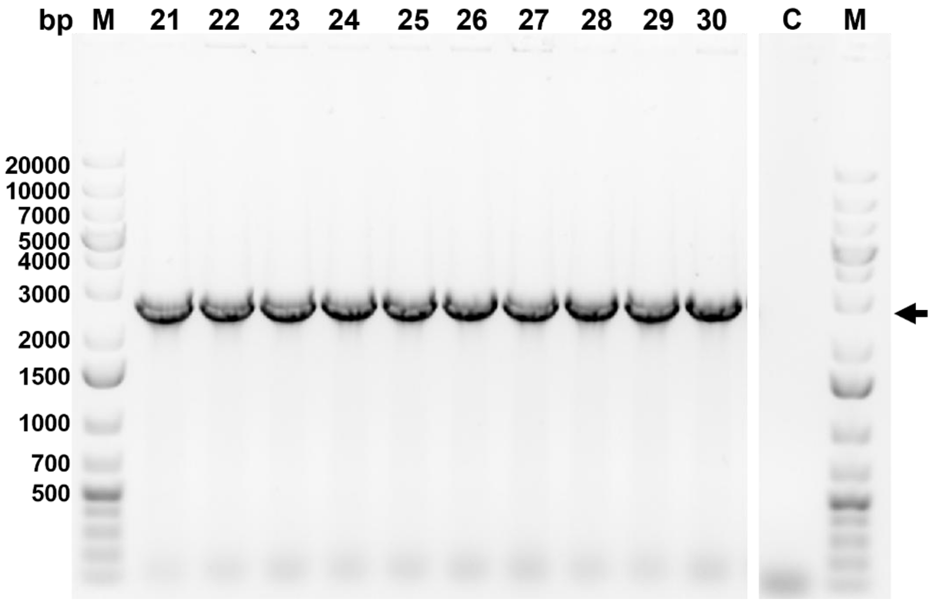

Figure S11: Verification of the integration of P_{GAP}-αMF-DAO-SE cassette plasmid into the *K. phaffii* genome by PCR. 1 % (w/v) agarose gels. M = Gene Ruler 1 kb Plus DNA Ladder. C = control (*K. phaffii* ATCC 76273). 21–30 = recombinant *K. phaffii* clones. The arrow indicates the expected DNA band for integration of the cassette plasmid into the *K. phaffii* genome.

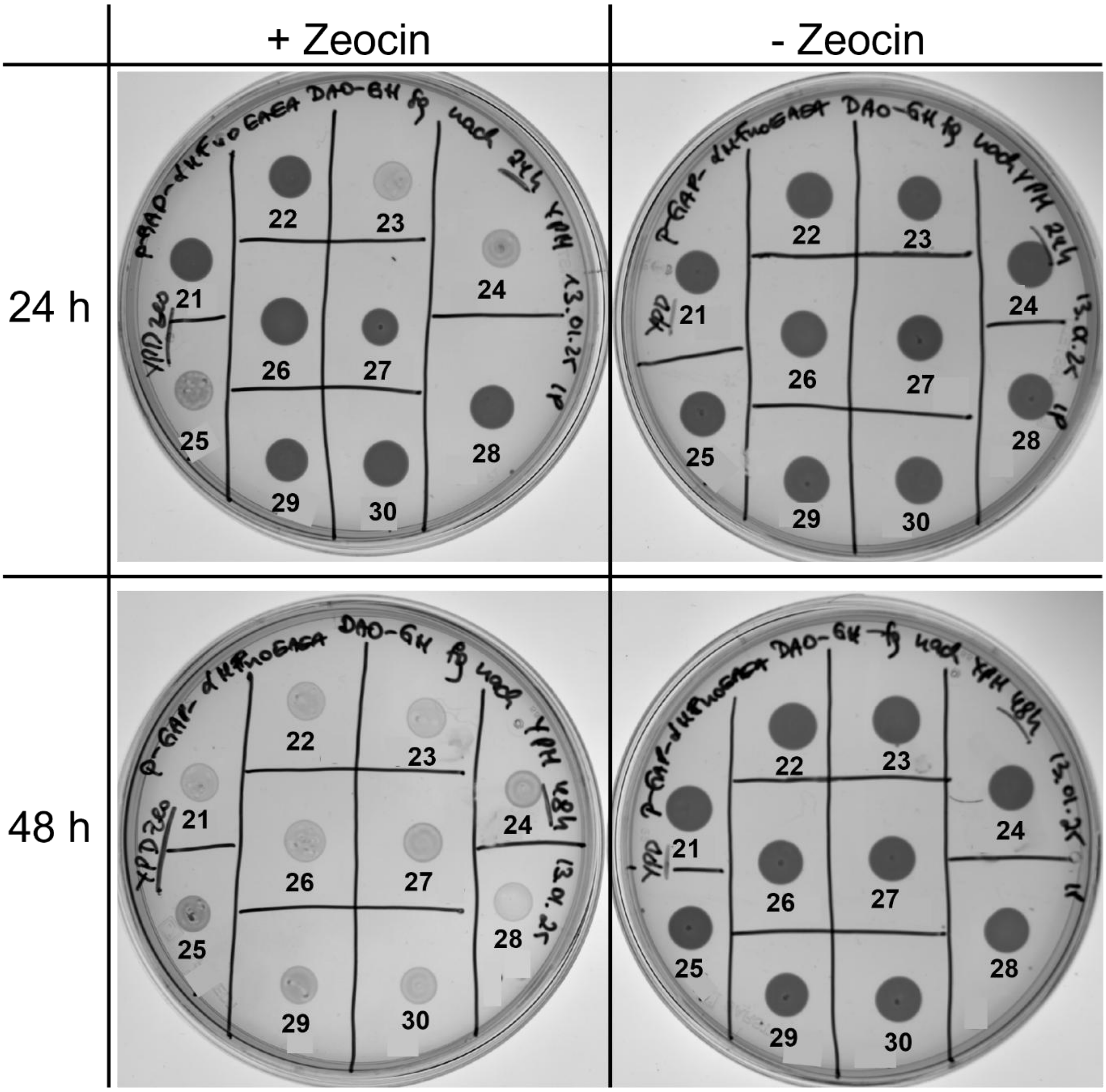

Figure S12: Agar plate screening to verify the excision of antibiotic resistance markers in recombinant *K. phaffii* clones. *K. phaffii* clones (21–30) with integrated P_{GAP}-αMF-DAO-SE cassette plasmid were incubated in methanol-containing medium for 24 and 48 h to induce Flp recombinase expression for marker excision. Isolated single colonies were spotted on agar plates with (+) and without (-) Zeocin. The agar plates were incubated for about 24 h at 30 °C. A total of ten recombinant *K. phaffii* clones were tested.

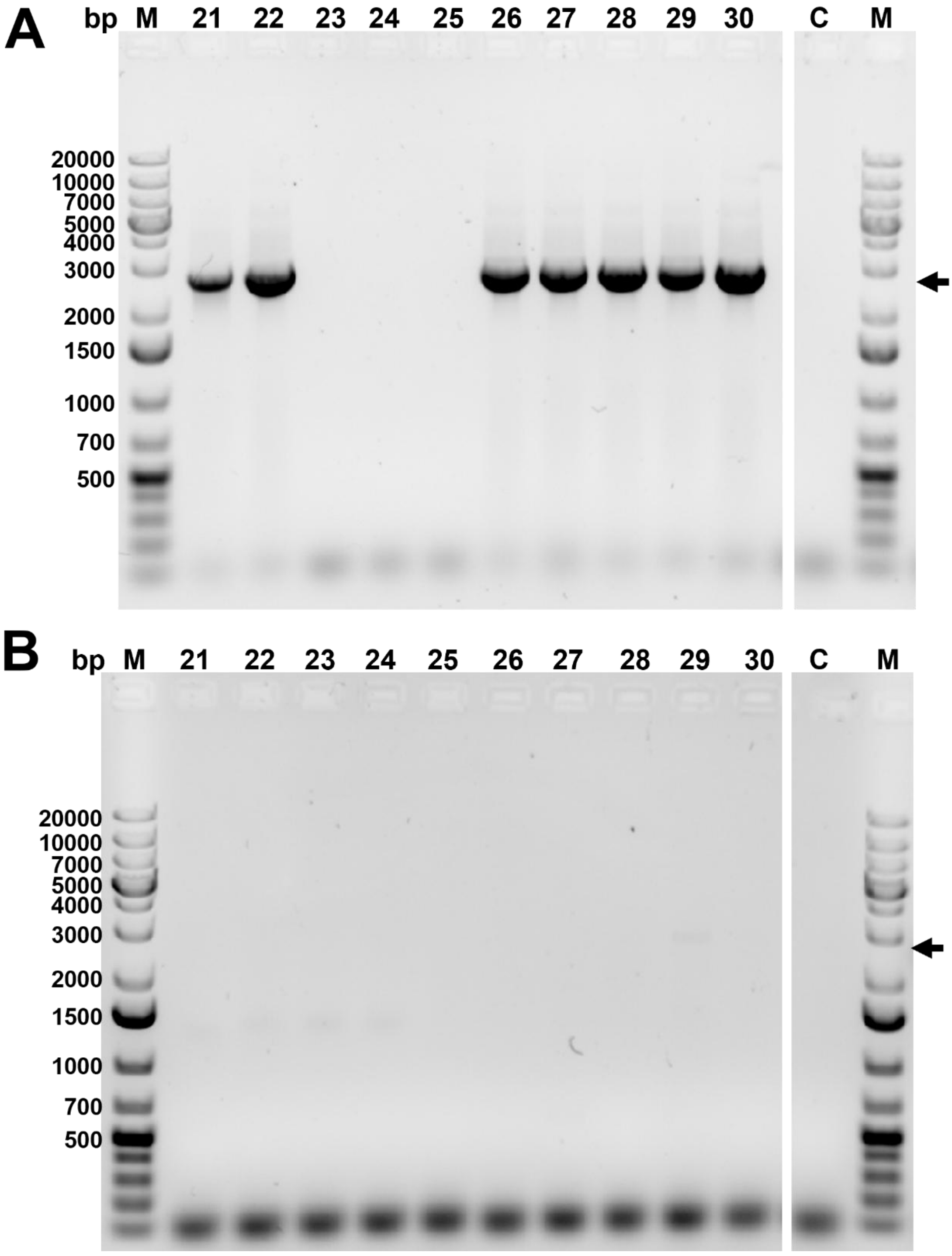

Figure S13: Verification of the antibiotic resistance marker excision in recombinant *K. phaffii* clones after 24 (A) and 48 h (B) incubation in medium containing methanol by PCR. 1 % (w/v) agarose gels. M = Gene Ruler 1 kb Plus DNA Ladder. C = control (*K. phaffii* ATCC 76273). 21–30 = recombinant *K. phaffii* clones (same as in Figure S12). A PCR product (indicated by arrow) was expected if antibiotic resistance markers were not excised; no PCR product was expected if antibiotic resistance markers were excised.

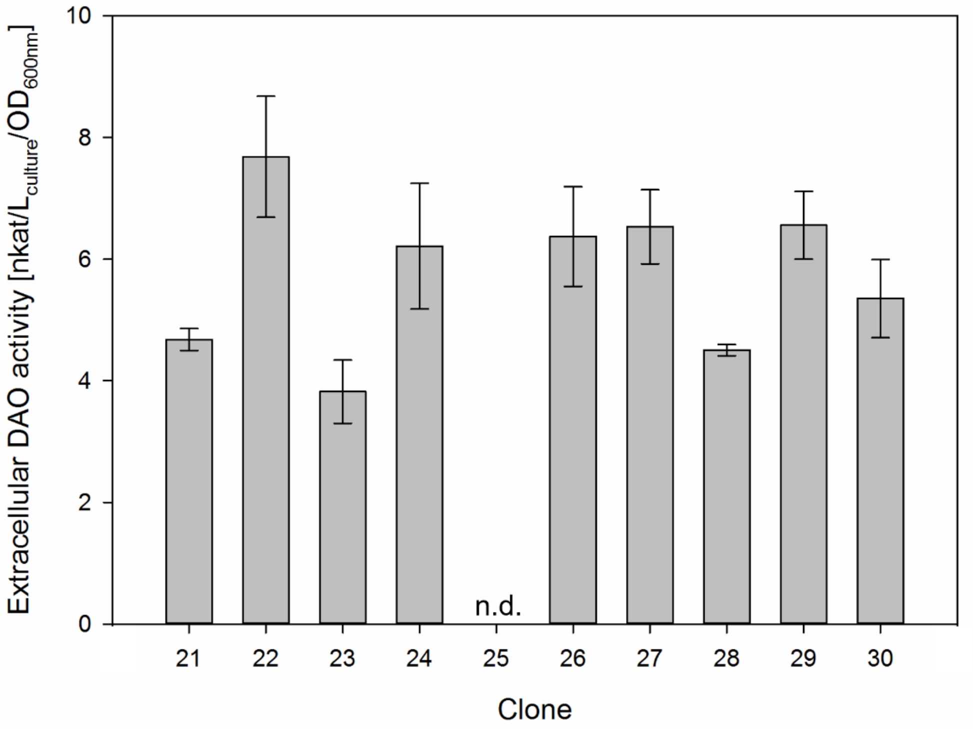

Fig. S14: Investigation of the extracellular DAO activity of antibiotic-resistance-free *K. phaffii* clones. Cultivation was done in tubes in a 5 mL working volume at 30 °C using YPD medium. The DAO activity was determined after 24 h of cultivation. n.d. = not detected.

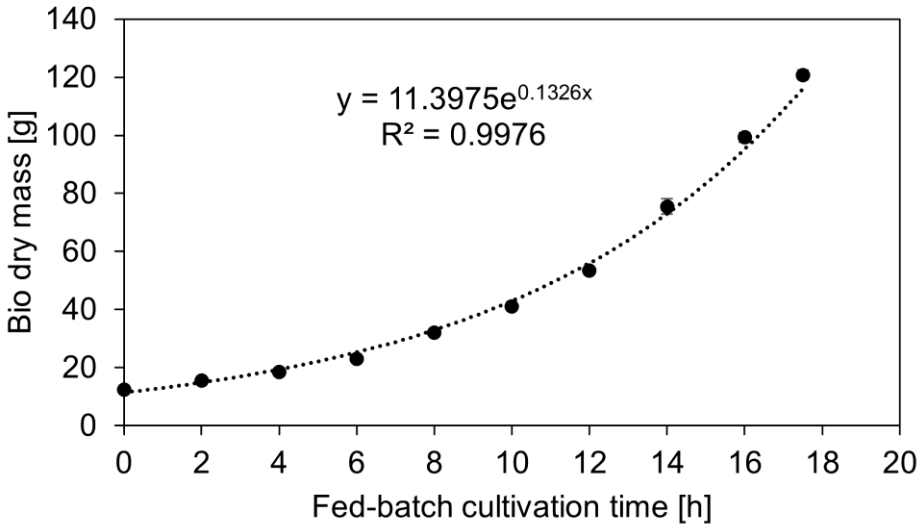

Figure S15: Total bio dry mass during the fed-batch bioreactor cultivation of antibiotic-resistance-free *K. phaffii* clone 22. BSM$_{glucose}$ medium, 0.5 L initial fermentation volume, 30 °C, pH 6. The specific growth rate was determined by fitting an exponential curve through experimental data.

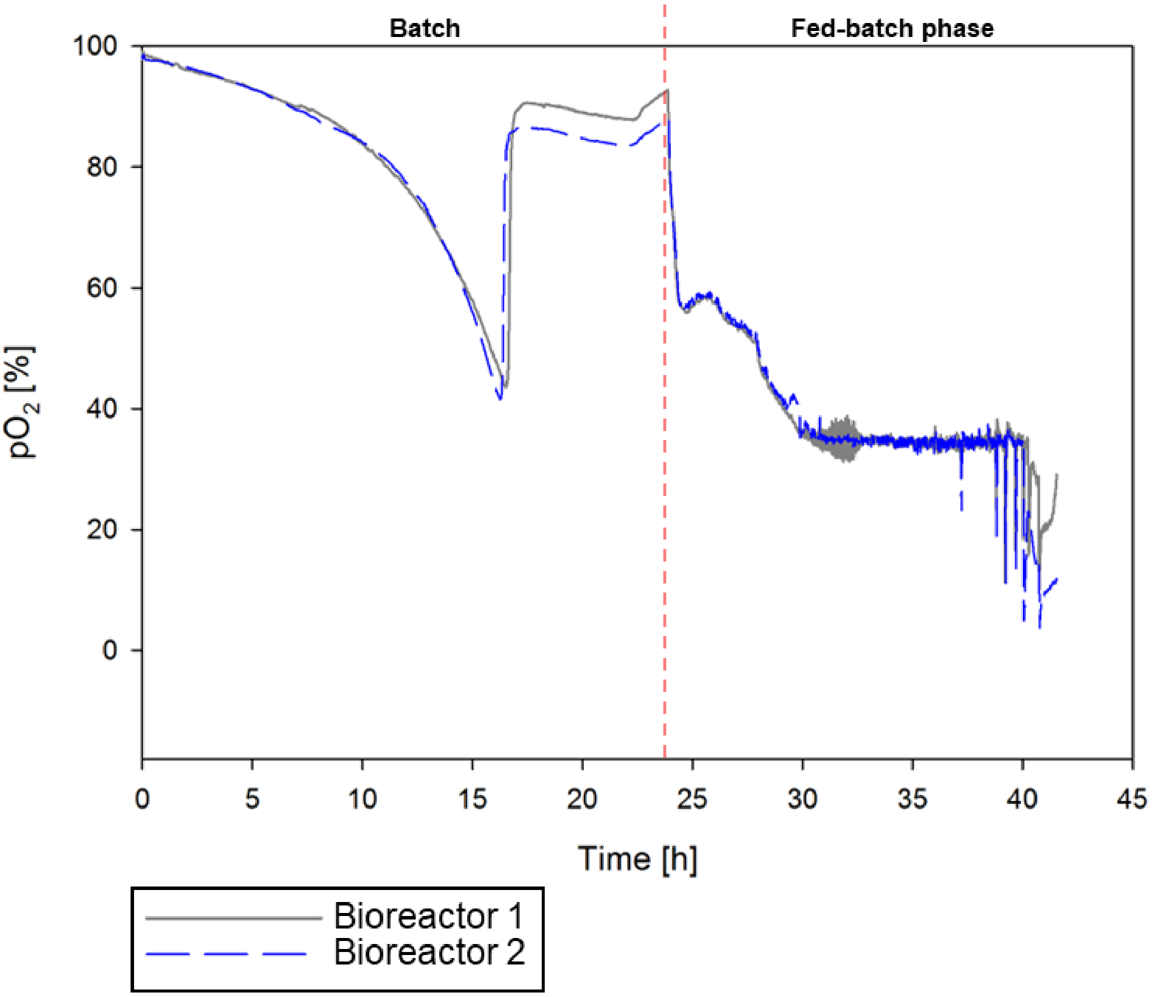

Figure S16: pO_2 profiles of biological duplicates during fed-batch bioreactor cultivations of antibiotic-resistance-free *K. phaffii* for secretory DAO-GH production. $BSM_{glucose}$ medium, 0.5 L initial fermentation volume, 30 °C, pH 6.

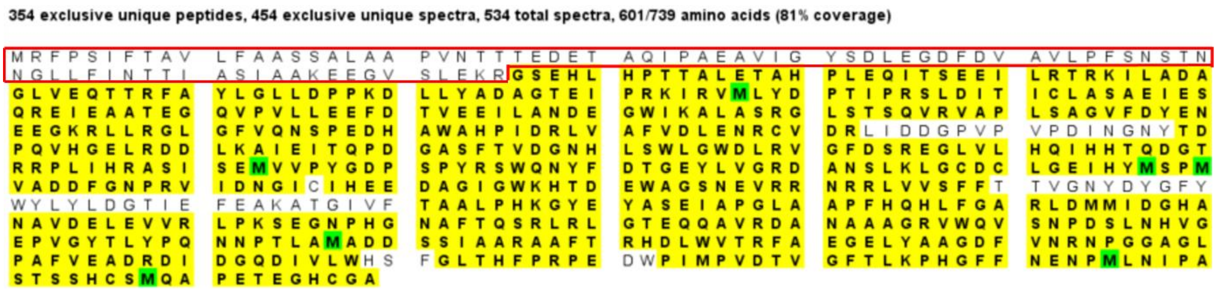

Figure S17: Mass spectrometry analysis of DAO-GH secreted by antibiotic-resistance-free *K. phaffii* clone. Sequences identified are highlighted in yellow, modifications in green. The αMF_noEAEA signal peptide is framed in red.

References

Bechtel A, Seitl I, Pross E, Hetzel F, Keutgen M, Fischer L (2024) Recombinant production of *Paenibacillus wynnii β*-galactosidase with *Komagataella phaffii*. Microb Cell Fact 23:263. https://doi.org/https://doi.org/10.1186/s12934-024-02544-5

Kettner L, Freund A, Bechtel A, Costa Català J, Fischer L (2025) A diamine oxidase from *Glutamicibacter halophytocola* for the degradation of histamine and tyramine in foods. Foods 14:3093. https://doi.org/10.3390/foods14173093

Lee ME, DeLoache WC, Cervantes B, Dueber JE (2015) A highly characterized yeast toolkit for modular, multipart assembly. ACS Synth Biol 4:975–986. https://doi.org/10.1021/sb500366v

Obst U, Lu TK, Sieber V (2017) A modular toolkit for generating *Pichia pastoris* secretion libraries. ACS Synth Biol 6:1016–1025. https://doi.org/10.1021/acssynbio.6b00337
